# ﻿Taxonomic study of *Palumbina* Rondani (Lepidoptera, Gelechiidae, Thiotrichinae) in Japan: biology, immature stages, and a new species

**DOI:** 10.3897/zookeys.1165.101983

**Published:** 2023-05-30

**Authors:** Khine Mon Mon Kyaw, Sadahisa Yagi, Johei Oku, Toshiya Hirowatari

**Affiliations:** 1 Entomological Laboratory, Graduate School of Bioresource and Bioenvironmental Sciences, Kyushu University, 744 Motooka, Nishi-ku, Fukuoka, 819-0395 Japan Kyushu University Motooka Japan; 2 Entomological Laboratory, Faculty of Agriculture, Kyushu University, 744 Motooka, Nishi-ku, Fukuoka, 819-0395 Japan Kyushu University Motooka Japan

**Keywords:** Chaetotaxy, host plants, larva, morphology, pupa

## Abstract

The genus *Palumbina* Rondani, 1876 belongs to the family Gelechiidae, which was until recently believed to comprise 26 species worldwide and mainly occurring in the Oriental region. Previously, only *P.pylartis* (Meyrick, 1908) had been recorded from this genus in Japan. In this study, five other species were identified. Four species were recorded for the first time in Japan, and another was newly described: *P.acerosa* Lee & Li, 2018; *P.grandiunca* Lee & Li, 2018; *P.macrodelta* (Meyrick, 1918), *P.operaria* (Meyrick, 1918) and *P.muraseae* Kyaw & Yagi, **sp. nov.** The host plant and larval feeding habits of *P.pylartis*, *P.acerosa*, *P.grandiunca*, and *P.muraseae* Kyaw & Yagi, **sp. nov.** were revealed. The immature stages of *P.grandiunca*, *P.pylartis*, and *P.muraseae* Kyaw & Yagi, **sp. nov.**, including larval and pupal morphology, were first documented for the genus in which the larval chaetotaxy of *P.pylartis* and *P.grandiunca* is well observed. In their larval chaetotaxy, the details of their interspecific variation in the relative position and length of some setae are described. The pupal morphology of the species examined in this study is almost identical to the genus *Thiotricha* except for different traits on the abdominal segments A7 and A10. The traits of larval chaetotaxy and pupal morphology are also discussed for the subfamily. Photographs of the adult, male and female genitalia, and information on their biology and immature stages are provided.

## ﻿Introduction

The genus *Palumbina* Rondani, 1876 has been treated as a member of Thiotrichinae based on morphological similarities (Karsholt et al. 2013). Recently, Lee et al. (2021) reviewed the phylogenetic relationships among the genera of this subfamily with extensive molecular analysis based on seven markers (COI, EF-1α, GAPDH, RpS5, CAD, Wg, and MDH) and 95 morphological characters for 47 ingroup and three outgroup taxa. The analyses in their study showed that those *Palumbina* species formed a strongly supported monophyletic group, including the type species *P.guerinii* (Stainton, 1858).

Although Rondani (1876) first established this genus, a better generic concept was redefined by Meyrick (1907, 1925a). This genus previously comprised 14 species, including small-to medium-sized moths (Sattler 1982; Walia and Wadhawan 2005). Recently, 12 new species have been described in China (Lee et al. 2018), and there are 26 species worldwide, mainly occurring in the Oriental region, especially in India and China. In contrast, only one species, *P.pylartis* (Meyrick, 1908) and two unidentified species have been recorded in Japan (Oku et al. 2018; Jinbo 2021).

The genus *Palumbina* is similar in appearance to the family Stathmopodidae, having long-bristled hind tibiae and lanceolate forewings and also showing a similar resting position (Meyrick 1925a,b; Robinson et al.1994; Sakamaki 2013). The larval feeding habits and host plants have been reported in *P.pylartis* as a petiole-miner on *Quercusmyrsinaefolia* Blume (Fagaceae) (Kuroko 1957; Sattler 1982). *Palumbinaguerinii* feeds on fruits, aphid-galls on leaves, or mine into the leaves of *Pistacia* sp. (Anacardiaceae) (Stainton [1857]; Sattler 1982). *Palumbinaglaucitis* (Meyrick, 1907) has been recorded feeding on leaves of *Mangiferaindica* L. (Anacardiaceae) (Sattler 1982). In addition, ovoviviparity is reported for the females of eight species of *Palumbina* (Lee and Li 2018).

A previous study in China (Lee et al. 2018) explored the diversity of Chinese *Palumbina* based on morphological characters and molecular data using the mitochondrial COI barcode region. The species groups were established with an identification key to Chinese *Palumbina*. However, studies on the biology and immature stages of this genus are still scarce. In Japan, studies on the genus *Palumbina* have been limited for several years. It is necessary to reveal the species diversity of Japanese *Palumbina* and investigate their immature stages.

In the present study, this genus was taxonomically reviewed to clarify the diversity of Japanese *Palumbina*, to review and explore their biology and larval chaetotaxy and describe their immature stages, including larval and pupal morphology.

## ﻿Materials and methods

The specimens used in this study were borrowed and examined from collections of universities and museums in Japan. Collection field trips were also performed in some localities throughout Japan between 2015 and 2021. Adults were collected using sweep nets unless otherwise indicated or light traps (**LT**). Larvae on the host plant were visually collected and reared in the laboratory (*Castanopsissieboldii* (Makino) Hatus. ex T.Yamaz. et Mashiba subsp. from Ito campus, Fukuoka in 2021, *Toxicodendronsuccedaneum* (L.) Kuntze from Aira River, Iriomotejima Is. in 2019 and *Distyliumracemosum* Siebold et Zucc. from Yaka, Okinawa Is. in 2021). For morphological observation, some of the last instar larvae and pupae were kept in 70–80% ethanol. The specimens were observed under a stereoscopic microscope (Nikon SMZ-U), and genitalia slides were prepared following Robinson (1976) after incubation in 10% KOH solution. Photographs of the male and female genitalia were taken using a biological microscope (Olympus BX43) with a digital camera (Olympus E-5). The images were combined by focus stacking using Zeren Stacker (2021).

The larvae were dissected for morphological analysis of the immature stages after incubation in 10% KOH. Larval and pupal morphologies were observed under a stereomicroscope (Nikon SMZ-U). The location of their genital opening confirmed the sex of the pupae. Larval chaetotaxy and pupal drawing were performed with a drawing tube attached to a biological microscope (Nikon Eclipse E400) and modified in Adobe Illustrator. Photographs of adults were taken using a digital camera (SONY α7R IV), and pupal morphology was observed using a stereomicroscope (Leica S8APO) with a digital camera (Canon EOS 7D).

The holotype and paratypes of *P.muraseae* sp. nov. and immature stages of *P.grandiunca*, *P.muraseae* sp. nov., and *P.pylartis* are deposited at the Entomological Laboratory, Faculty of Agriculture, Kyushu University, Fukuoka, Japan (**ELKU**). The other examined specimens were deposited in the following institutions: Hikosan Biological Experimental Facility, Kyushu University, Soeda, Japan (**HBEF**); Laboratory of Systematic Entomology, Hokkaido University, Sapporo, Japan (**SEHU**); Entomological Laboratory, Osaka Prefecture University, Osaka, Japan (**OPU**); Entomological Laboratory, Kagoshima University, Kagoshima, Japan (**KGU**); and National Museum of Nature and Science, Tsukuba, Japan (**NSMT**). Type locality (**TL**) and Type depository (**TD**) are indicated in the synonymic list of each species. Type specimens of previous works are deposited in the following museums: Natural History Museum, London**(NHMUK)** and Insect collection, Nankai University, Tianjin (**NKU**).

### ﻿Terminology

The expressive terminology was based on Lee et al. (2018) and that of the male and female genitalia, followed Klots (1970), Hodges (1974), Huemer and Karsholt (1999) and Lee et al. (2018). Larval chaetotaxy followed Hinton (1946) and Stehr (1987). The pupal morphological character description followed Mosher (1916), Patočka and Turčáni (2005). The scientific names of the plants follow Yonekura and Kajita (2003) by identifying the host plants based on Hayashi (2014) and Ohkawa and Hayashi (2016).

## ﻿Results

### ﻿Taxonomy

#### ﻿Genus Palumbina

Taxon classificationAnimaliaLepidopteraGelechiidae

Rondani, 1876

573A8B07-12C7-5B57-8DED-7902C61DCFEA

Palumbina Rondani, 1876. Type species: Palumbinaterebintella Rondani, 1876 (= Palumbinaguerinii (Stainton, [1857])), by monotypy.
Thyrsostoma Meyrick, 1907. Type species: Thyrsostomaglaucitis Meyrick, 1907, by monotypy. Synonymized by Sattler 1982: 25.

#### ﻿ Palumbina pylartis

Taxon classificationAnimaliaLepidopteraGelechiidae

(Meyrick, 1908)

A3F49742-A757-5397-A6D4-0E6FFA2DD785

Figs 1A, B , 6A , 7A , 8A , 9A , 10 , 14 , 15 , 18 , 19 Japanese name: Minami-mongin-hoso-kibaga

Thiotricha pylartis Meyrick, 1908: 441. TL: Assam, India. TD: NHMUK.
Thyrsostoma pylartis : Meyrick 1925a: 100; Gaede 1937: 301; Inoue 1954: 67 (partim); Clarke 1969: 487; Moriuti 1982: 281, pl. 13–57 (partim); Kanazawa and Hepper 1992: 70.
Palumbina pylartis : Sattler 1982: 25; Sakamaki 2013: 298 (partim).

##### Material examined.

Japan – **Honshu** [Gifu] • 1♀; 20 Oct. 1919; gen. slide no. KM–333; ELKU. – [Wakayama] • 1♂; Kozagawa; 11 Jun. 1970; host: *Castanopsiscuspidata*; T. Kumata; gen. slide no. KM–390; SEHU. • 2♀♀; same data except 9, 13 Jun. 1970; gen. slide no. KM–377; SEHU. – **Kyushu** [Fukuoka] • 1♀; Nishi-ku Fukuoka, Ito Campus; 27 Apr. 2017 larva; 1 Jun. 2017 em.; host: *Castanopsissieboldii*; T. Hirowatari, S. Yagi, C. Tsuji & K.M.M. Kyaw leg.; ELKU. • 1♂, 1♀; same locality, 5 May 2020 larva; 23 May 2020 em.; Host: *Castanopsissieboldii*; S. Yagi leg.; ELKU. • 1♀; same locality, 3 Jul. 2021, LT; S. Yagi leg.; ELKU. [Kagoshima] • 1♂; Yakushima Is., Hirauti; 19 Sep. 1978; Y. Arita leg.; gen. slide no. KM–329; NSMT. • 1♂; Nakanoshima Is., Mt. Otake; 12 Sep. 2018; K. Sakagami leg.; gen. slide no. KM–330; ELKU. – **Ryukyus** [Kagoshima] • 1♀; Amamioshima Is., Oosima-gun, Uken-son, Mt. Akatsuchi; 5 Oct. 2020, LT; J. Oku leg.; ELKU. • 1♂; Tokunoshima Is., Yonama Amagi–cho; 11 Jul. 2016; S. Yagi leg.; gen. slide no. KM–383; ELKU. – [Okinawa] • 1♂; Kunigami district, Oogimi, Oshikawa area; 27 Mar. 2021, LT; J. Oku leg.; ELKU. • 2♀♀; Ishigakijima Is., Mt. Bannadake; 4–7 Apr. 2001; T. Ueda leg.; gen. slide no. KM–424; OPU. • 1♀; Iriomotejima Is., Airagawa forest road, Taketomi Yaeyama; 26 Aug. 2020; Y. Hisasue leg.; ELKU.

**Figure 1.**
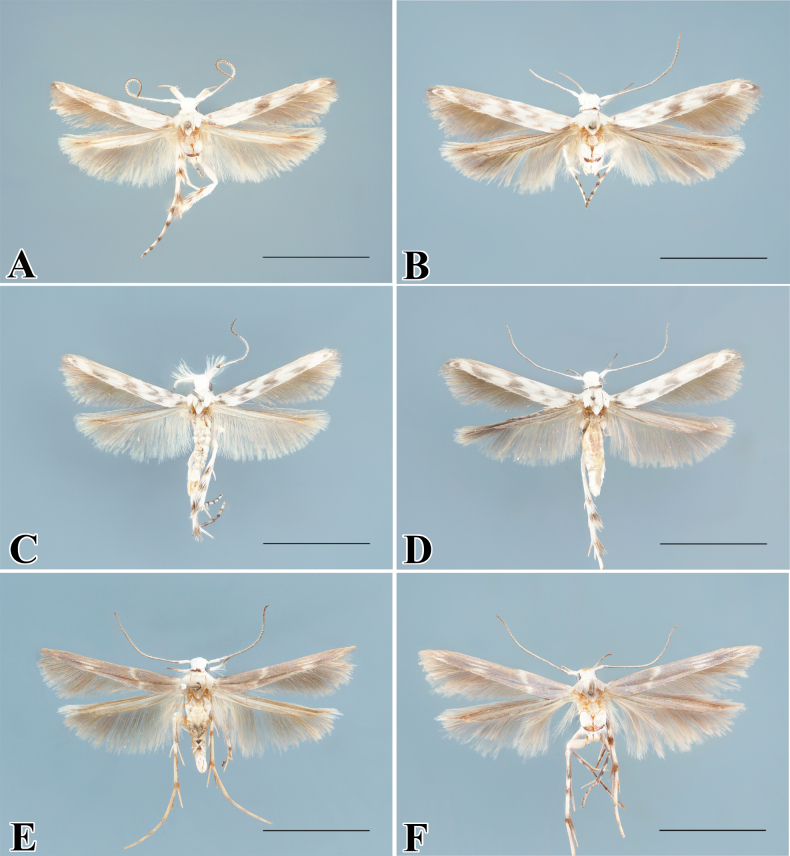
Adults of *Palumbina* spp. **A***P.pylartis*, male **B** ditto, female **C***P.acerosa*, male **D** ditto, female **E***P.grandiunca*, male **F** ditto, female. Scale bars: 4.0 mm.

For the diagnosis of the adult and the detailed description of the adult and genitalia, see Lee et al. (2018).

##### Description.

**Larva** (Figs 10G, H, 14, 15). Length 3.5–3.6 mm (*n* = 2). Head subglobular, yellowish brown with blackish pigmentations on ocellar area and anterior margin of labrum. Body pale yellow in early instars and yellowish brown in late instars. Prothoracic shield yellowish brown, with blackish brown on caudal margin. Thoracic legs short, pale yellow (Fig. 15A). Pinacula more or less rounded, blackish brown on T1–T3, A1, A2, A8, and A9; paler on the remaining abdominal segments. Anal shield heavily sclerotized, yellowish brown (Figs 14D, 15D). Anal fork present, deeply emarginated posteriorly, forming two lateral lobes (Figs 14E, 15D). Anal prolegs armed with many minute spines on dorsal surface. Crochets in a circle, uniordinal, 10–14 in number on ventral prolegs (Fig. 15B), 6–8 on anal proleg (Fig. 15C).

**Figure 2.**
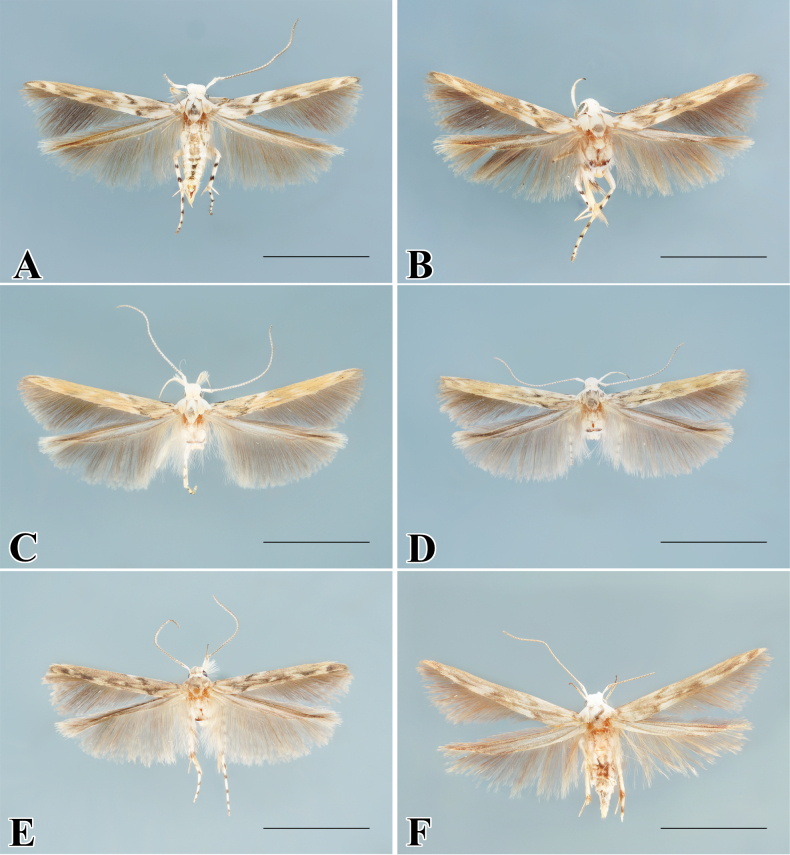
*Palumbinaoperaria* (wing marking variation) adult. **A, C, E** male **B, D, E** female. Scale bars: 4.0 mm.

***Chaetotaxy*** (Fig. 14). ***Head*** (Fig. 14A, B). Epicranial suture shorter than frontoclypeus, AF1 ~ ½ length of AF2; C2 slightly longer than C1; P1 dorsolateral to AF1, ~ 5× longer than P2; P2 dorsolateral to AF2 and above P1; MD1–MD3 setae forming nearly in a line at the posterior margin of the head capsule, MD1 slightly anteroventral to MD2 and MD3; mouthparts semi-hypognathous; genal area with six stemmata, forming a semicircular pattern; A1 dorsoanterior to stemma-3, slightly shorter than A3; A2 dorsolateral to A1 and shorter than A1 and A3; L1 dorsoposterior to stemma-1; distance between L1 from A3 slightly longer than distance between A3 from A2; S1 below stemma-3, short as A2; S2 longer than S1 and S3, near the opening of the stemmatal semicircle; S3 slightly shorter than S1 and ventroposterior to stemma-6; SS1 near a mandibular condyle, the same length as SS2; SS2 between SS1 and SS3; SS3 ~ 3× longer than SS1 and SS2; MGa present, close to MG1. Mandible with five teeth (Fig. 14C).

**Figure 3.**
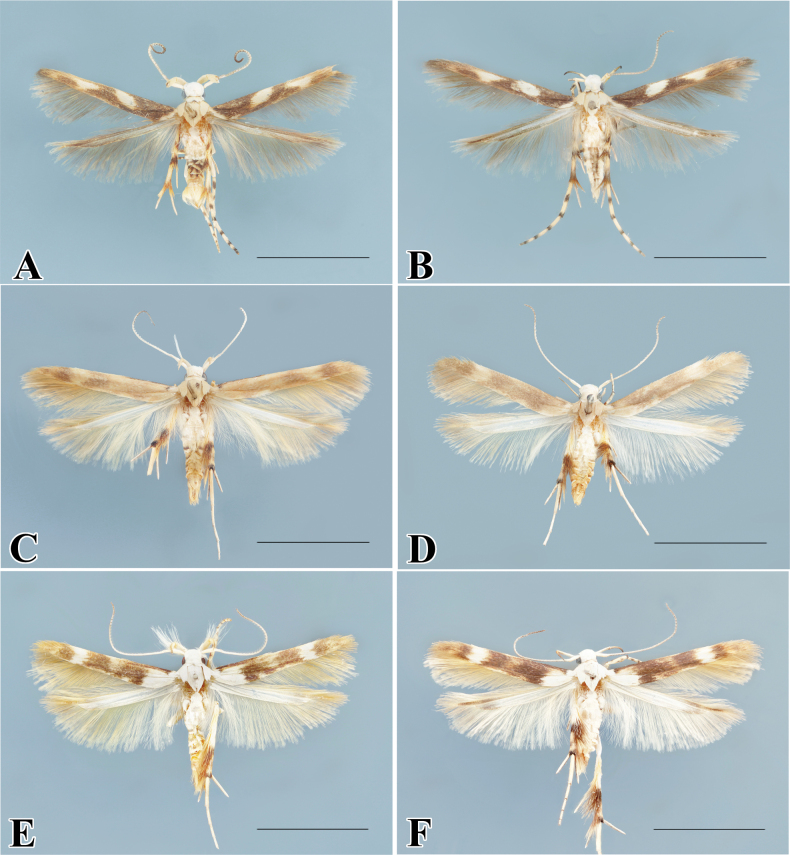
Adults of *Palumbina* spp. **A***P.macrodelta*, male **B** ditto, female **C***P.muraseae* sp. nov., male, holotype **E** male, paratype **D**, **F** ditto, female, paratypes. Scale bars: 4.0 mm.

***Thorax*.** Prothorax (Fig. 14F): shield with SD1 ventrolateral to XD1 and XD2, all three along anterior margin; XD2 less than 2½ distance from XD1 than from SD1; XD1 ~ 2× longer than XD2; SD2 and D1 ca. equal in length, both setae ~ 2½–3× length of SD1 and D2; SD2 ca. twice the distance from XD2 than from SD1; L-group tri-setose on the same pinaculum, anteroventral to spiracle; L1 longest; L2 and L3 short, ca. equal in length; SV-group bi-setose on the same pinaculum; SV1 ~ 2–2½ × longer than SV2; MV1 absent; MV2 approximate to anterolateral coxal margin; V1 approximate mesoposterior coxal margin. Mesothorax and metathorax (Fig. 14F): D and SD group arranged in a vertical line; D1 and D2 on same pinaculum, D2 ~ 3½–4× length of D1; SD1 and SD2 on the same pinaculum, SD1 ~ 3½–4× length of SD2; MD1 anteroventral to D2; MSD1 in line with MSD2, anterior to SD2; MSD2 anterior to SD1; L-group trisetose; L1 ~ 2½–3× length of L2, each on separate pinaculum; pinaculum of L2 slightly anterior to SD group pinaculum; L3 slightly longer than L2, vertical line with SV1; MV1, MV2, and MV3 anterior to coxa; MV2 near to anterolateral coxal margin, MV3 slightly above V1.

***Abdomen*** (Fig. 14F). A1 and A2 with D2 ~ 3½–4× longer than D1; D1 dorso-anterior to D2; MD1 slightly ventral to D1 and D2; SD1 ca. equal in length to D2; SD2 minute, anteroventral to SD1; SD1 above spiracle; L-group tri-setose; L1 and L2 on separate pinaculum, L2 in vertical line with SD1 and spiracle; L1 ~ 3½–4× longer than L2 and L3; L3 slightly longer than L2; SV-group bi-setose on A1, SV1 ~ 2½–3× longer than SV2 and on same pinaculum; SV-group tri-setose on A2, with SV1 and SV2 on the same pinaculum, SV3 on separate pinaculum (not shown); MV3 dorso-anterior to V1. A3–A6 as in A2, except D2 dorsoposterior to D1 and SV1–SV3 on separate pinaculum; each segment bearing a pair of protuberant prolegs; planta bearing uni-ordinal, uniserial crochets in a circle (Fig. 15B). A7 as in A3–A6 except with SV-group bi-setose on same pinaculum. A8 as A7 except: SD1 ~ 1/3× length of D2 and in vertical line with D2; SD2 remote from spiracle and not vertical to MD1; minute SD2 anteroventral to SD1 and spiracle; SD1 pinaculum slightly anterodorsal to spiracle; spiracle dorsal to all spiracles on A1–A7; L1 ~ 3½–4× length of L2 and below spiracle; L2 and L3 on separate pinaculum; L2 anteroventral to L1 and in vertical line with D2 and SD1; L3 ventral to L2; SV and V group uni-setose. A9 as above except with D1 ventral to D2, D2 ~ 2× longer than D1; D-group and MD1 on same pinaculum; MD1 in horizontal line with D2; SD1 absent; L-group bi-setose on same pinaculum, L1 ~ 3½–4× longer than L2.

**Pupa** (Figs 18, 19). Length 3.5–4.2 mm (*n* = 3). Cylindrical, yellowish brown, dark brown before emergence. Head semi-globular. Vertex with many minute spines. Prothorax with a pair of more or less wedge-shaped projections on the dorsolateral corner of tergite (Fig. 19A, D). Antennae and forewing reach the midway or near posterior margin of A6. Maxilla (galea) basally broad, gradually narrowing and extending to the posterior margin of A4. Prothoracic legs extending to A2; mesothoracic legs extending to near posterior margin of A4; metathoracic legs extending to midway or near posterior margin of A7. A5–A10 movable. A5 and A6 with a transverse row of tergal spinules directed posteriorly on the anterior margin in males (Fig. 18A, C) and with a transverse row of dot-like spinules in females (Fig. 18D, F). A7 with a transverse row of tergal spinules, directed posteriorly on the anterior margin in both males and females (Fig. 18A, D). Sternite A7 with a pair of oval pads armed with a row of spinules directed anteriorly in females (Fig. 18E) but absent in males (Fig. 18B). A10 delineated with a row of short spinules along its outer margin posteriorly in both males and females (Fig. 18C, F), apically with three pairs of hooked setae on ventral surfaces of A9 and A10.

##### Distribution.

Japan (Honshu, Kyushu, Ryukyu), China, Taiwan, and India.

##### Host plant.

*Castanopsiscuspidata* (Thunb.) Schottky (new host record), *C.sieboldii* (Makino) Hatus. ex T. Yamaz. et Mashiba (new host record) (Fagaceae).

##### Biology

(Fig. 10). We found one preserved specimen of this species with a label noting that it feeds on *Castanopsiscuspidata* (Fagaceae) and a case of the leaf of its host plant. We found that the larvae fed on another host plant, *C.sieboldii*. In the latter host plant, the larva first makes a small hole (Fig. 10C, red arrow) to enter and feed on the plant tissue in the midrib of the leaf, and this hole also seems to eliminate its feces. After the larva gradually develops by feeding inside the midrib, it leaves the midrib by creating another small exit hole (Fig. 10C, black arrow). After leaving the mine, the larva crawls towards the upper tip of the leaf, cuts transversely from its outer margin to the inner part of the leaf (Fig. 10D, E), and then makes a shelter with it. The larva usually consumes the leaf by leaning halfway from its shelter. Subsequently, it cuts the leaf around the radius and reaches a small and regular shape to construct a portable case (Fig. 10F, G). After that, a number of these several small leaf pieces (5–7 pieces, *n* = 6) were accumulated and stacked into a compact, approximately circular leaf case until the final instar larva (Fig. 10I). The final larva fixes its case (Fig. 10J), and pupation occurs inside its case (Fig. 10K). The adult emerges by leaving the pupal exuvia (Fig. 10L). The resting posture of the adult is similar to that of the stathmopodid species, keeping its hindlegs upwards (Fig. 9A). This species overwinters in the adult stage, and adults are collected throughout the year (Sakamaki 2013).

**Figure 4.**
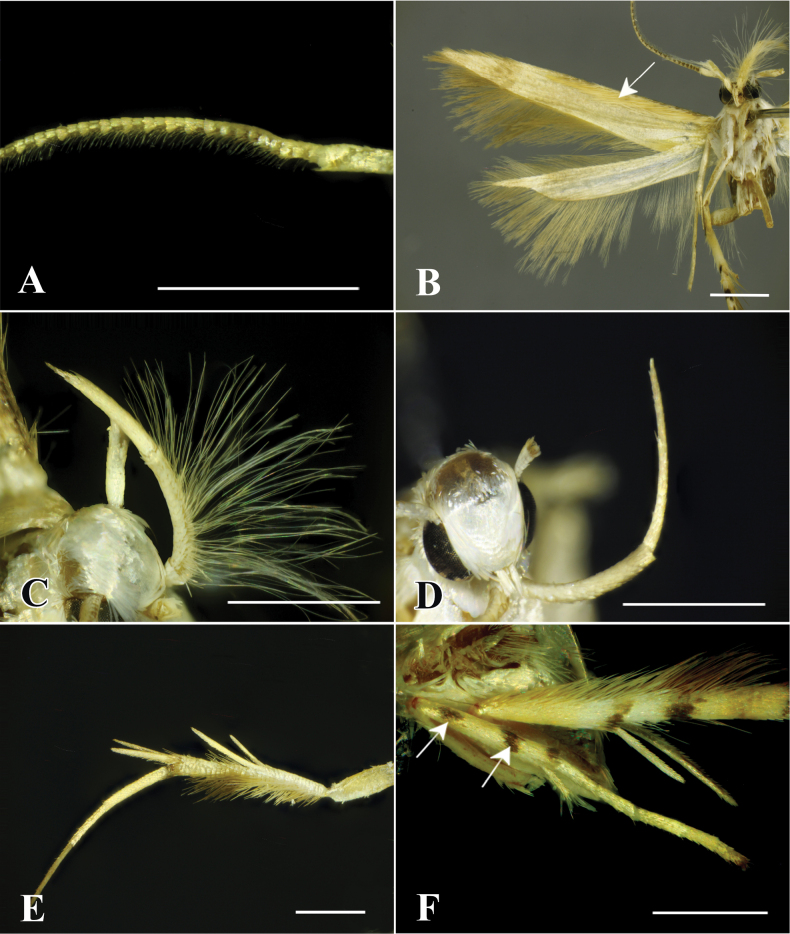
External morphological characters of *P.muraseae* sp. nov. **A** male antenna **B** the ventral surface of the wing. The arrow indicates expansible hair pencils beneath costa **C** labial palpus of male modified with long expansible hair pencil **D** labial palpus of female **E** hind leg **F** mid-leg. The arrows indicate two black spots. Scale bars: 1.0 mm.

##### Remarks.

The larva of *P.pylartis* was reported as a petiole-miner on the host plant *Quercusmyrsinaefolia* Blume (Fagaceae) on Mt. Hikosan, Fukuoka Pref. (Kuroko 1957). However, we could not find any specimens at ELKU and HBEF that were shown as the depositories in that study, and all specimens collected on Mt. Hikosan in the present study were identified as *P.acerosa*. Therefore, we conclude that the previous host record of “*Thyrsostomapylartis*” reported by Kuroko (1957) was misidentified as *P.pylartis* because they are very similar to each other in external morphological characters and in the female genitalia.

According to previous records (e.g., Inoue 1954; Moriuti 1982; Sakamaki 2013), *P.pylartis* is distributed from Honshu to the Ryukyus in Japan. However, *P.pylartis* is not common in Honshu and Kyushu, whereas *P.acerosa* is expected in this region. Thus, previous distributional records must be reviewed.

#### ﻿ Palumbina acerosa

Taxon classificationAnimaliaLepidopteraGelechiidae

Lee & Li, 2018

FA80892F-22D3-5876-B11F-49C119249801

Figs 1C, D , 6B , 7B , 8B , 9B , 11 Japanese name: Kita-mongin-hoso-kibaga

Palumbina acerosa Lee & Li, 2018: Lee et al. 2018: 27, fig. 81. TL: Guizhou, China; TD: NKU.
Thyrsostoma pylartis : Inoue 1954: 67 (partim); Kuroko 1957: 7; Moriuti 1982: 281, pls 13–57 (partim).
Palumbina pylartis : Sakamaki 2013: 298 (partim).

##### Material examined.

Japan – **Honshu** [Kanagawa] • 1♂; Yamakita-cho, Lake. Tanzawa; 4 Jun. 2016; Y. Kitajima leg.; KGU. –[Shizuoka] • 1♀; Atami-shi, Himensosawa Koen; 10 Sep. 2002; T. Hirowatari, B. W. Lee, H. Mizukawa, S. Takaki & K. Tateiwa leg.; gen. slide no. KM–426; OPU. • 1♀; Kannami-cho, Kannami-Genseirin; 10 Sep. 2002; T. Hirowatari, B. W. Lee, H. Mizukawa, S.Takaki & K. Tateiwa leg.; OPU. • 1♀; Shibakawa-cho, Inago Iriyama; 20 Aug. 1999; T. Mano leg.; OPU. • 1♀; same data except 28 Aug. 1999; gen. slide no. KM–425; OPU. – [Aichi] • 2♂♂; Kitashidara-gun, Uradani (900m); 17 Sep. 1977; Y. Arita leg.; NSMT. • 1♂; Toyota, Mt. Rokusho-san; 28 Oct. 1976; Y. Arita leg.; NSMT. • 1♂; Higashihagihira, Asahi-cho, Sahara shrine; 30 May 1998; T. Mano leg.; OPU. • 1♂; Asahi-cho, Sakakino; 280m; 20 Jun. 1998; T. Mano leg.; OPU. – [Mie] • 4♂♂, 1♀; Iga-shi; Host: *Quercusglauca*; 8 Oct. 2019 em.; Y. Arita leg.; ELKU. – [Osaka] • 1♀; Tottori; 7 Jun. 1994; S. Kosino leg.; OPU. • 1♂; same data except 10 Sep. 1998; OPU. • 1♂; Mt. Inunaki; 22 Nov. 1998; S. Kosino leg.; OPU. – [Wakayama] • 2♂♂, 2♀♀; Kozagawa; 22–28 Oct. 1974 em.; Host: *Quercusglauca*; T. Kumata leg.; SEHU. – **Shikoku** [Ehime] • 1♂, 3♀♀; Matsuyama-Castle areas, Matsuyama-shi; 23, 30 Aug. 2016, LT; J. Oku leg.; KGU. – [Kochi] • 1♂; Iwaidani; 16 Apr. 1964; S. Moriuti leg.; OPU. – **Kyushu** [Fukuoka] • 1♀; Nishi-ku Fukuoka, Ito Campus; 27 Jun. 2017; T. Hirowatari, S. Yagi, C. Tsuji & K.M.M. Kyaw leg.; ELKU. • 1♂, 1♀; same data except 17 Aug. 2018, LT; S. Yagi leg.; ELKU. • 1♂, 2♀♀; same data except 18 Sep. 2019, LT; J. Oku leg.; ELKU. • 1♂, 1♀; same data except 30 Oct. 2019, LT; K.M.M. Kyaw leg.; ELKU. • 2♀♀; same data except 20 Mar. 2021; S. Yagi leg.; ELKU. • 1♀; Mt. Sefuri, Itaya, Sawara-Ward, Fukuoka-City; 2 Jun. 2019; S. Tomura, K. Sasaki leg.; ELKU. • 1♂; Mt. Hikosan, Soeda, Tagawa-Distinct; 25 Sep. 2014, LT; S. Yagi leg.; ELKU. • 1♀; same data except; 27 Jul. 2015, LT; ELKU. • 1♂; same data except 1 Jul. 2016; gen. slide no. KM–320.; ELKU. – [Oita] • 1♂; Yufu-shi, Meisuinotaki 850m, Mt. Kuro; 13 Jun. 2015, LT; S. Yagi leg.; gen. slide no. KM–321.; ELKU. – [Kumamoto] • 1♂; Tatsudayama; 29 Mar. 1960; T. Kawarabata leg.; ELKU. – [Miyazaki] • 1♂; Higashiusuki-gun, Shiiba-son, Research Forest; 4 Jun. 2018; K. Uemori leg.; ELKU. – [Kagoshima] • 1♀: Tanegashima Is., Nishinoomote; 13 Jun. 1965; T. Kumata leg.; gen. slide no. KM–376; SEHU. – **Ryukyus** [Kagoshima] • 2♂; Amamioshima Is., Santaro-toge (Sumiyo-son); 30 Apr. 1999; T. Saito leg.; KM–319; OPU. • 2♀♀; Amamioshima Is., Mt. Yuwan, Uken-son; 6 Jul. 2016 larva; 20 Jul. 2016 em.; Host: *Quercusglauca*; S. Yagi leg.; gen. slide no. KM–381; ELKU. • 2♀♀; same data except 6 Jul. 2016 larva; 25 Jul. 2016 em.; gen. slide no. KM–332; SY–197; ELKU. – [Okinawa] • 1♀; Okinawajima Is., Ookuni-rindo (Kunigami-son); 26 Mar. 2002; T. Saito leg.; gen. slide no. KM-331; ELKU.

**Figure 5.**
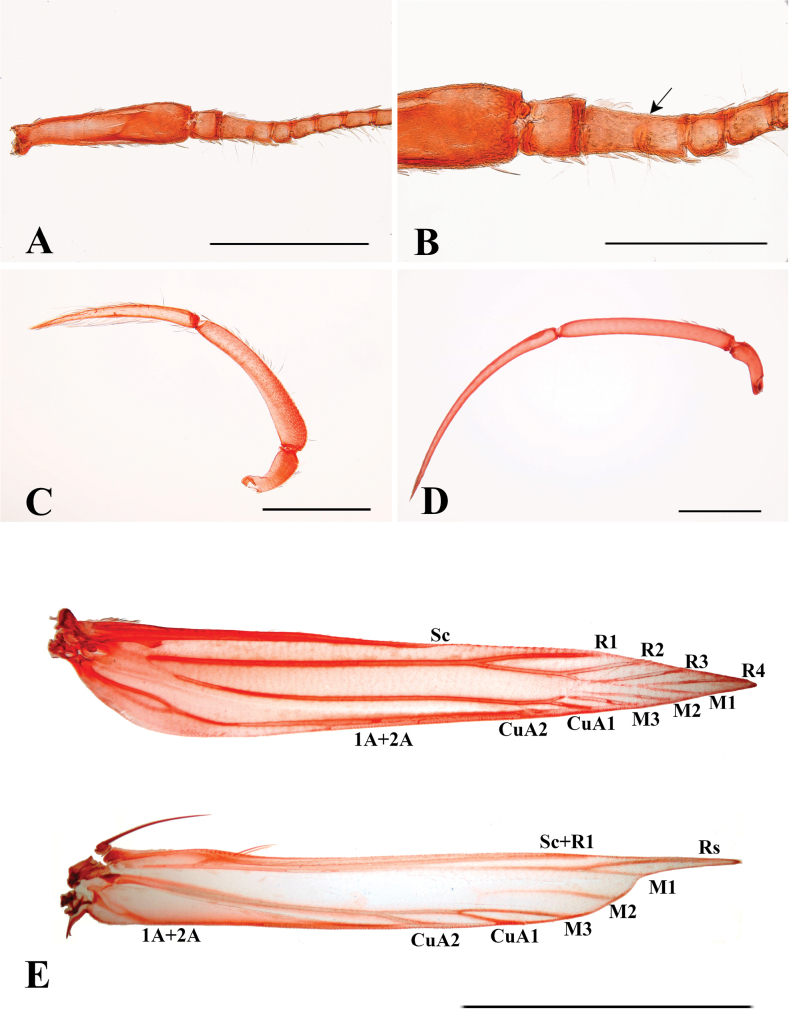
Antenna, labial palpus, and wing venation of *P.muraseae* sp. nov. **A, B** basal part of the male antenna. The arrow indicates unseparated flagellomeres I and II **C** labial palpus of male **D** labial palpus of female **E** male fore and hind wing venation. Scale bars: 0.40 mm (**A, C, D**); 0.20 mm (**B**); 1.0 mm (**E**).

For the diagnosis and detailed description of the adults and genitalia, see Lee et al. (2018).

**Figure 6.**
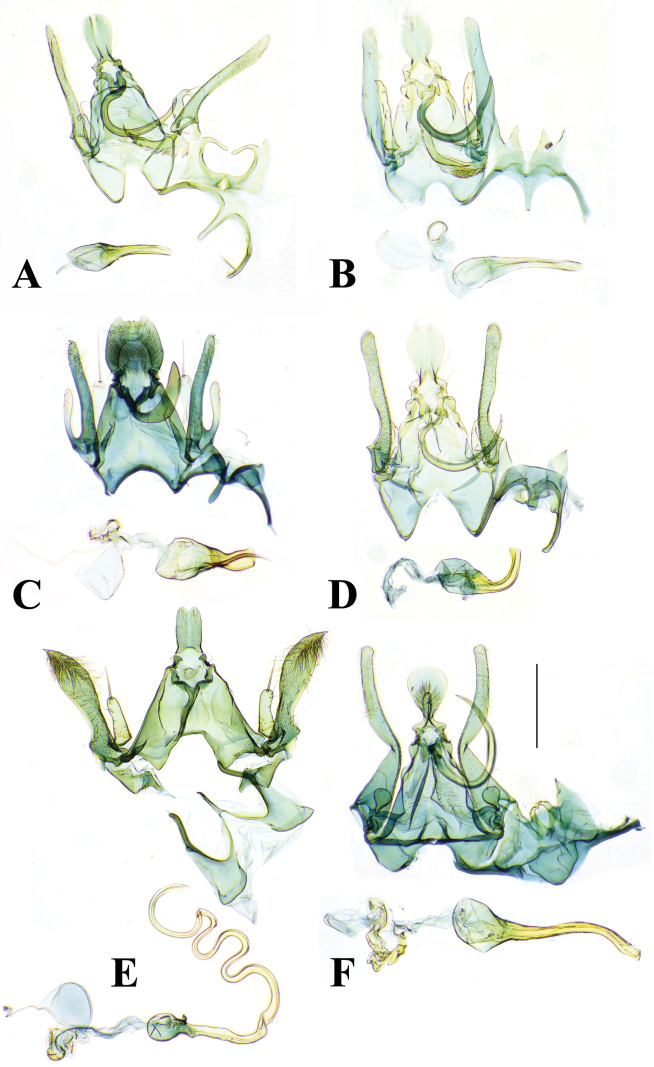
Male genitalia of *Palumbina* spp. **A***P.pylartis*, genitalia slide no. KM–383 **B***P.acerosa*, genitalia slide no. KM–320 **C***P.grandiunca*, genitalia slide no. SY1454 **D***P.operaria*, genitalia slide no. KM–389 **E***P.macrodelta*, genitalia slide no. SY1455 **F***P.muraseae* sp. nov., holotype, genitalia slide no. KM–374. Scale bars: 0.40 mm.

##### Distribution.

Japan (Honshu, Shikoku, Kyushu, Ryukyu), China, Korea (Lee and Jeun 2022), and Taiwan (Lee et al. 2018). The distribution overlaps that of *P.pylartis* but tends to be found in the northern regions.

##### Host plant.

*Quercusmyrsinaefolia* Blume (Kuroko 1957, see the remarks of *P.pylartis*), *Q.glauca* Thunb. (new host record) (Fagaceae).

##### Biology.

The larval biology is very close to that of *P.pylartis* except for the host plants. We found young larvae on the host plant *Quercusglauca* (Fagaceae) on Amamioshima Island, behaving as a miner in the midrib of young leaves. This late-instar larva made a portable case by cutting the young leaf into small pieces, mainly around the upper portion (Fig. 11A, B).

The resting posture of the adult is similar to that of stathmopodid species, keeping their hindlegs upwards (Fig. 9B).

#### ﻿ Palumbina grandiunca

Taxon classificationAnimaliaLepidopteraGelechiidae

Lee & Li, 2018

879DE2AD-A2B0-52A0-B546-8480E9E8AE62

Figs 1E, F , 6C , 7C , 8C , 9C , 12 , 16 , 17 , 20 , 21 Japanese name: Hazenoki-ginhoso-kibaga

Palumbina grandiunca Lee & Li, 2018: Lee et al. 2018: 15, fig. 68. TL: Hainan, China. TD: NKU.
Palumbina sp. 1: Oku et al. 2018: 58, fig. 46.

##### Material examined.

Japan – **Honshu** [Hyogo] • 1♂; Inaba (Hidaka-tyo); 11 Jun. 1994; T. Saito leg.; gen. slide no. KM–305; OPU. – **Ryukyus** [Kagoshima] • 1♀; Amamioshima Is., Uken-son, Yuwandake; 21 Jul. 2003; T. Saito leg.; OPU. • 1♂; same locality; 19 Jun. 2014; S. Sameshima leg.; KGU. • 1♂; same data except 7 May 2015; gen. slide no.KM–304; KGU. • 1♂; same data except 18 May 2015; gen. slide no.KM–306; KGU. • 1♂; Amamioshima Is., Uken-son, Akatuti-yama; 18 Aug. 2012; S. Sameshima leg.; KGU. • 1♂; same data except 8 Sep. 2012; KGU. • 1♂; same data except 18 Aug. 2012; KGU. • 1♂; same data except 21 May 2013; KGU. • 1♂; same data except 12 Jun. 2013; gen. slide no. KM–303; KGU. • 2♂♂; same data except 25 May 2015; gen. slide no. KM–308; KGU. • 1♀; same data except 26 May 2015; gen. slide no. KM–310; KGU. • 1♂; same data except 27 May 2015; KGU. • 1♂; same data except 1 Jun. 2015; KGU. • 2♀♀; Amamioshima Is., Sumiyo-son, Kamiya; 23 May 2015; S. Sameshima leg.; KGU. • 1♂; Amamioshima Is., Buren; 14 Sep. 2014; S. Sameshima leg.; gen. slide no. KM–302; KGU. • 1♂; Amamioshima Is., Setouti-to-cho, Mt Yui-dake; 30 Sep. 2014; S. Sameshima leg.; gen. slide no. KM–307; KGU. • 1♂; Tokunoshima Is. San, Tokunoshima; 12 Jul. 2016, LT; S. Yagi leg.; ELKU. [Okinawa] • 1♂; Okinawajima Is., Benoki, Kunigami-son, Mt. Nishime 340m; 3 Aug. 2015, LT; S. Yagi leg., genitalia slide no. SY1454; ELKU. • 1♂; Okinawajima Is., Sate, Kunigami-son, Mt. Terukubi 300m; 28 May 2015, LT; S. Yagi leg.; ELKU. • 1♂; same data except 17 Mar. 2017; gen. slide no. KM–309; ELKU. • 1♀; Okinawajima Is., Yona, Kunigami-son; 6 May 2000; T. Saito leg.; gen. slide no. KM–312; OPU. • 1♂; Okinawajima Is., Hentona, Kunigami-son; 15 Mar. 2017, LT; S. Yagi leg.; ELKU. • 1♀, Okinawajima Is., Hizi, Kunigami-son; 3 Aug. 2002; T. Saito leg., gen. slide no. KM–311; OPU. • 1♂; Ishigakijima Is., Takeda-rindo; 5 Jul. 2017; S. Yagi leg.; ELKU. • 1♀; Iriomotejima Is., Aira River; 26 Jun. 2019 larva; 22 Jul. 2019 em.; Host: *Toxicodendronsuccedaneum*; K.M.M. Kyaw leg.; gen. slide no. SY1453; ELKU. • 2♂♂, 1♀; Iriomotejima Is., Sonai Forestry Park; 27 Jun. 2019 larva; 16–17 Jul. 2019 em.; Host: *Toxicodendronsuccedaneum*; K.M.M. Kyaw leg.; ELKU. • 1♂; Iriomotejima Is., Shirahama; 8 May 2012, LT; T. Hirowatari, S. Kobayashi, K. Nakatsuka, T. Yoshida leg.; OPU.

**Figure 7.**
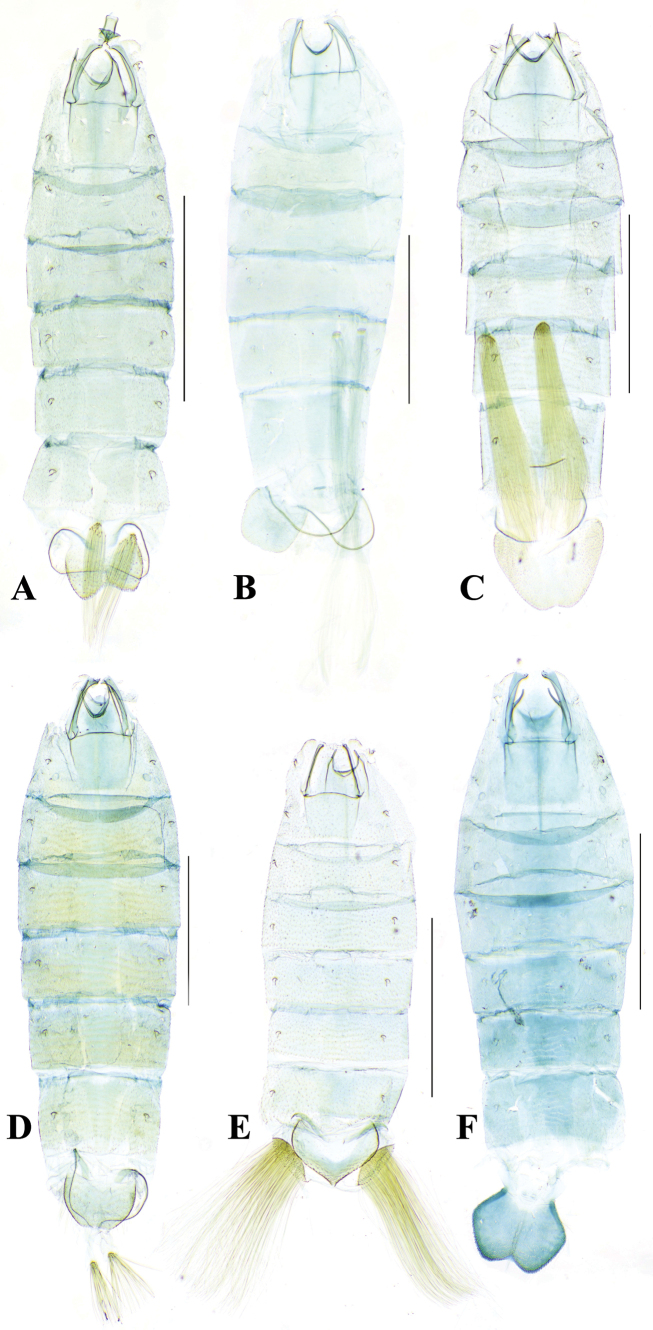
Abdominal segments of *Palumbina* spp. **A***P.pylartis*, slide no. KM-383 **B***P.acerosa*, slide no. KM–320 **C***P.grandiunca*, slide no. SY1454 **D***P.operaria*, slide no. KM–389 **E***P.macrodelta*, slide no. SY1455 **F***P.muraseae* sp. nov., holotype, slide no. KM–374. Scale bars: 1.0 mm.

For the diagnosis and detailed description of the adults and genitalia, see Lee et al. (2018).

##### Description.

**Larva** (Figs 12I, 16, 17). Length ~ 3.0 mm (*n* = 6), slender. Head subglobular; yellowish brown, with blackish pigmentations on ocellar area and on anterior margin of labrum. Body pale yellow in early instars and yellowish brown in late instars. Prothoracic shield yellowish brown, with blackish brown on caudal margin. Thoracic legs short, pale yellowish brown. Pinaculum more or less rounded, blackish brown on T1–T3, A1, A2, A8, and A9; paler on remaining abdominal segments. Anal shield heavily sclerotized, yellowish brown (Figs 16D, 17D). Anal fork present, deeply emarginated, forming two lateral lobes (Figs 16E, 17D). Anal prolegs armed with many minute spines on dorsal surface (Fig. 17C). Crochets uni-ordinal, 13–17 in number on ventral prolegs (Fig. 17B), 9–11 on anal prolegs (Fig. 17C).

**Figure 8.**
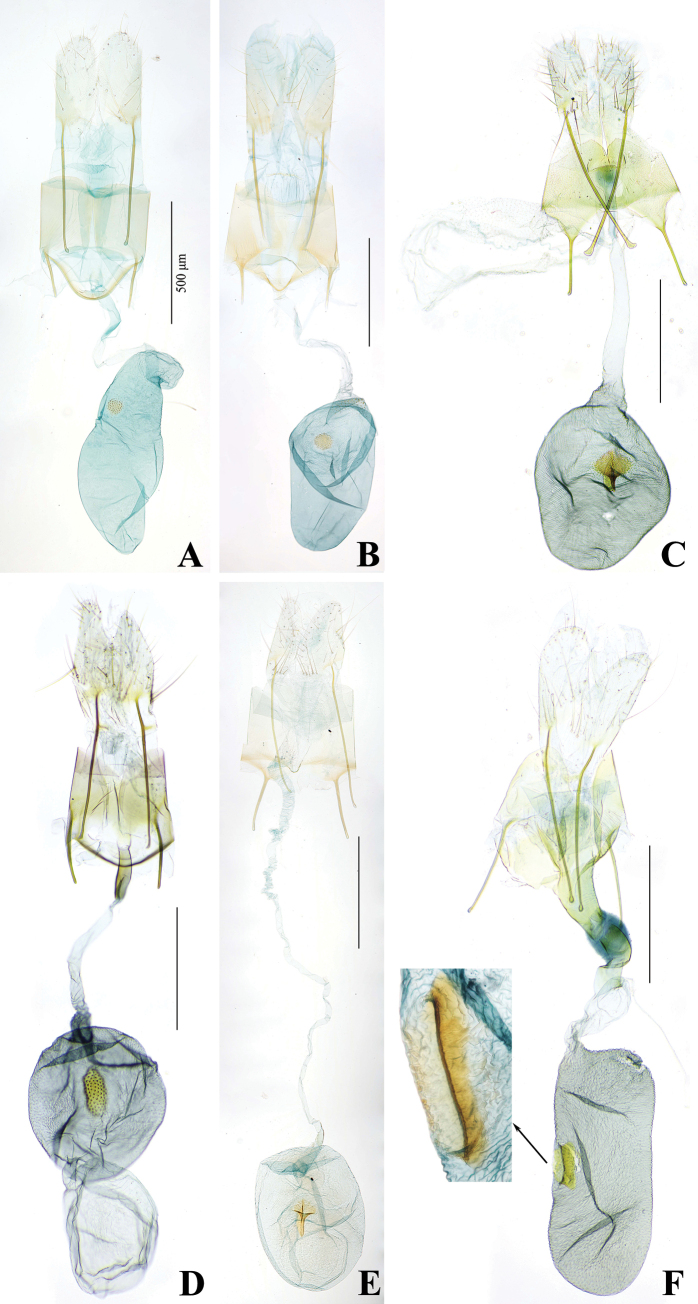
Female genitalia of *Palumbina* spp. **A***P.pylartis*, genitalia slide no. KM–330 **B***P.acerosa*, genitalia slide no. KM–381 **C***P.grandiunca*, genitalia slide no. SY1453 **D***P.operaria*, genitalia slide no. *Palumbina* No. 1 **E***P.macrodelta*, genitalia slide no. KM–328 **F***P.muraseae* sp. nov., whole genitalia, genitalia slide no. KM–348 and signum, genitalia slide no. KM–427. Scale bars: 0.50 mm.

***Chaetotaxy*** (Fig. 16). ***Head*** (Fig. 16A, B): epicranial suture shorter than fontoclypeus; AF1 ~ ½ length of AF2; C2 slightly longer than C1; P1 dorsolateral to AF1, ~ 5× longer than P2; P2 dorsolateral to AF2 and above P1; MD1–MD3 setae forming nearly in a line at the posterior margin of head capsule, MD1 slightly anteroventral to MD2 and MD3; mouthparts semi-hypognathous; genal area with six stemmata, forming a semicircular pattern; A1 dorso-anterior to stemma-3, slightly shorter than A3; A2 dorsolateral to A1 and shorter than A1 and A3; L1 dorsoposterior to stemma-1; distance between L1 from A3 slightly longer than distance between A3 from A2; S1 below stemma-3 and short as A2; S2 longer than S1 and S3, near the opening of the stemmatal semicircle; S3 slightly shorter than S1 and ventroposterior to stemma-6; SS1 near mandibular condyle, same length as SS2; SS2 between SS1 and SS3; SS3 ~ 3× longer than SS1 and SS2; MGa present, close to MG1. Mandible with five teeth (Fig. 16C).

**Figure 9.**
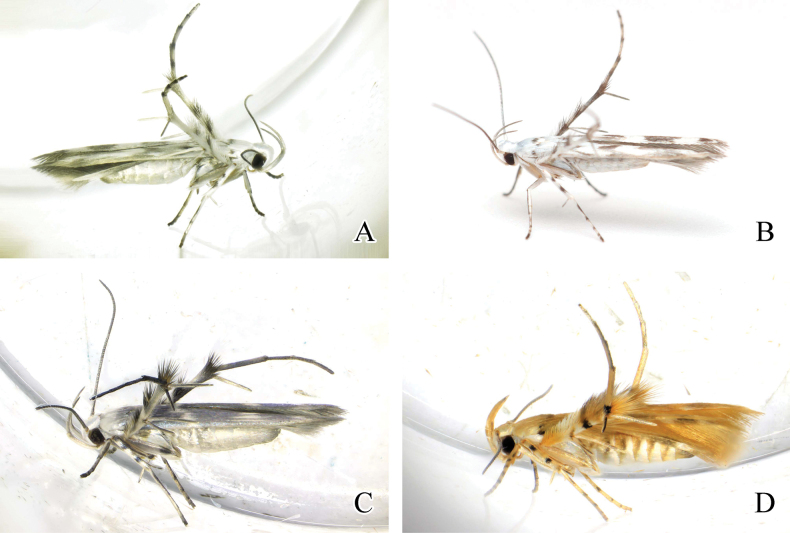
Resting position of adult *Palumbina* spp. **A***P.pylartis*, female **B***P.acerosa*, female **C***P.grandiunca*, female **D***P.muraseae* sp. nov., male.

***Thorax*** (Fig. 16F): Prothorax: Shield with SD1 ventrolateral to XD1 and XD2, all three along anterior margin; XD2 equal distance from XD1 and SD1; XD1 ~ 2× longer than XD2; SD2 and D1 ca. equal in length, both setae less than ~ 2½–3× length of SD1 and D2; SD2 less than 1½ distance from XD2 than from SD1; L-group tri-setose on the same pinaculum, anteroventral to spiracle; L1 longest; L2 and L3 short, ca. equal in length; SV-group bi-setose on the same pinaculum; SV1 ~ 2–2½ × longer than SV2; MV1 absent; MV2 approximate to anterolateral coxal margin; V1 near to mesoposterior coxal margin. Mesothorax and metathorax (Fig. 16F): D1 and D2 on the same pinaculum; SD1 and SD2 on the same pinaculum; all arranged in a vertical line; D2 ~ 3½–4 length of D1; SD1 ~ 3½–4× length of SD2; MD1 anteroventral to D2; MSD1 in a line with MSD2, anterior to SD2; MSD2 anterodorsal to SD1; L-group tri-setose; L1 and L2 on the same pinaculum, L1 ~ 2½–3× length of L2, slightly anterior to D- and SD-group pinaculum; L3 slightly longer than L2, in a vertical line with SV1; MV1, MV2, and MV3 anterior to coxa; MV2 approximate to anterolateral coxal margin, MV3 slightly above V1.

**Figure 10.**
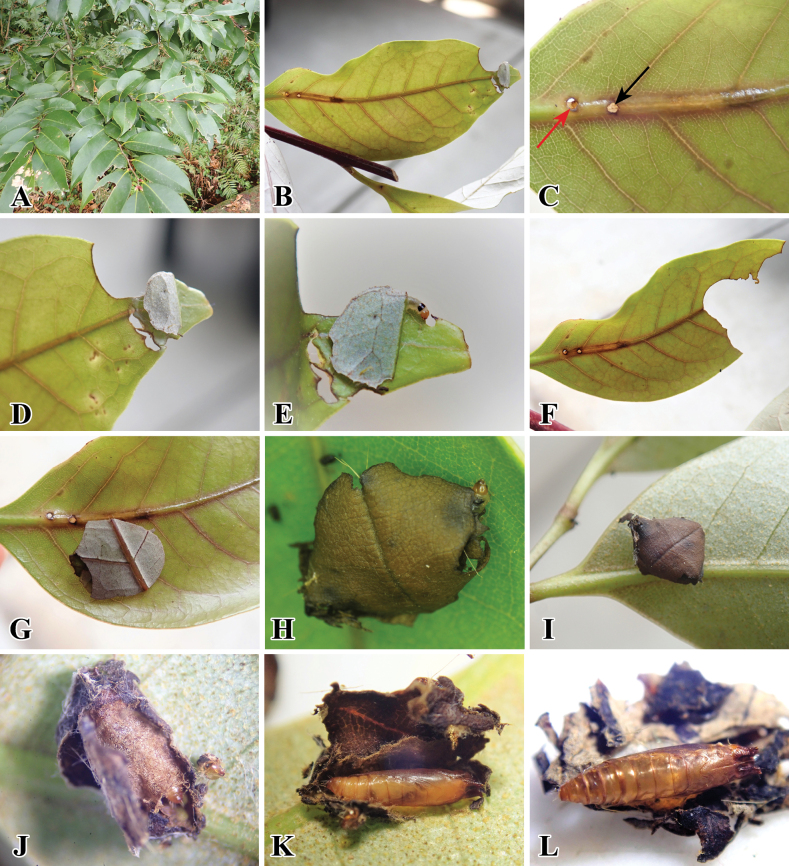
*Palumbinapylartis* and its host plant in Fukuoka Pref. **A** host plant: *Castanopsissieboldii* (Fagaceae) **B** infected leaf **C** small hole at the base of the midrib of leaf (Upper surface) **D** leaf tip cut by larva **E** larva protruding from its leaf shelter **F** leaf cut by larva **G** newly leaf case made by larva **H, I** dried leaf case on upper and lower leaf surface **J** pupa in a cocoon **K** pupa **L** pupal exuvia left by an emerged adult.

***Abdomen*** (Fig. 16F). A1 and A2 (not indicated) with D1 dorso-anterior to D2; D2 ~ 3½–4× longer than D1; MD1 slightly ventral to D1 and D2; SD1 above spiracle, ca. equal in length with D2; SD2 minute, anteroventral to SD1 and on different pinaculum; SD2 minute, anteroventral to SD1; L-group tri-setose; L1 and L2 on same pinaculum below spiracle, ventroposterior to SD group; L1 ~ 3½–4× longer than L2 and L3; L3 slightly longer than L2; SV-group on same pinaculum, bi-setose on A1 and tri-setose pinaculum on A2; SV1 ~ 2½–3× longer than SV2 on A1; MV3 dorso-anterior to V1. A3–A6 as in A2, except D2 dorsoposterior to D1; each segment bearing a pair of protuberant prolegs; planta bearing uni-ordinal, uniserial crochets in a circle (Fig. 17B). A7 as in A2 or A6 except with SV-group bi-setose and V1 ventral to SV pinaculum. A8 as A7 except SD1 in vertical line with D2; minute SD2 anteroventral and below spiracle; SD1 pinaculum slightly anterior to spiracle; spiracle dorsal to all spiracles on A1–A7; L1 ~ 3½–4× length of L2 and below spiracle; L2 and L3 on separate pinaculum; L2 anteroventral to L1 and in vertical line with D2 and SD1; L3 ventral to L2 and in vertical line with L2; SV and V group uni-setose. A9 as above except with D1 ventral to D2 and on same pinaculum; D2 ~ 2× longer than D1; MD1 slightly anteroventral to D2; hair-like SD1 present; L-group bi-setose on same pinaculum, L1 ~ 3½–4× longer than L2.

**Figure 11.**
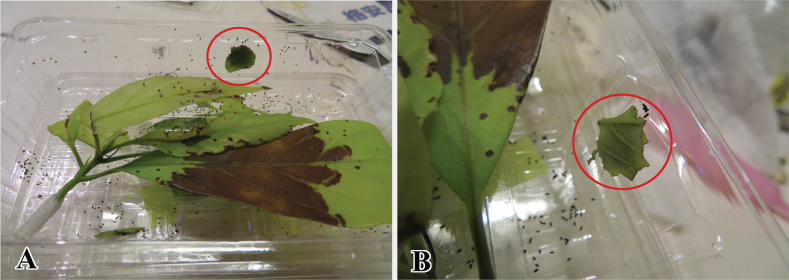
*Palumbinaacerosa* feeding on host plant *Quercusglauca* (Fagaceae) in Amamioshima Is. **A**, **B** larval leaf cases.

**Pupa** (Figs 20, 21). **Female.** Length 3.9–4.2 mm (*n* = 3). Cylindrical, color yellowishbrown; dark brown before emergence. Head semi-globular. Vertex with many minute spines. Prothorax with a pair of triangular projections on dorsolateral corners of tergite (Fig. 21A). Antennae reaching near the posterior margin of A5. Forewing reaching near the anterior margin of A6. Maxilla (galea) basally broad, gradually narrowing and extending to A4. Prothoracic legs extending to A2; mesothoracic legs reaching A4; metathoracic legs extending to the posterior margin of A7. A5–A10 movable. A5 and A6 with a transverse row of dot-like spinules on the anterior margin (Fig. 20C). A7 with a transverse row of tergal spinules directed posteriorly at the anterior margin (Fig. 20A). Sternite A7 with a pair of oval pads armed with a row of spinules directed anteriorly (Fig. 20B). A10 delineated with a row of short spinules at the outer margin posteriorly (Fig. 20A), apically with three pairs of hooked setae on ventral surfaces of A9 and A10, without true cremaster.

##### Distribution.

Japan (Honshu, Ryukyus) and China.

##### Host plant.

**Figure 12.**
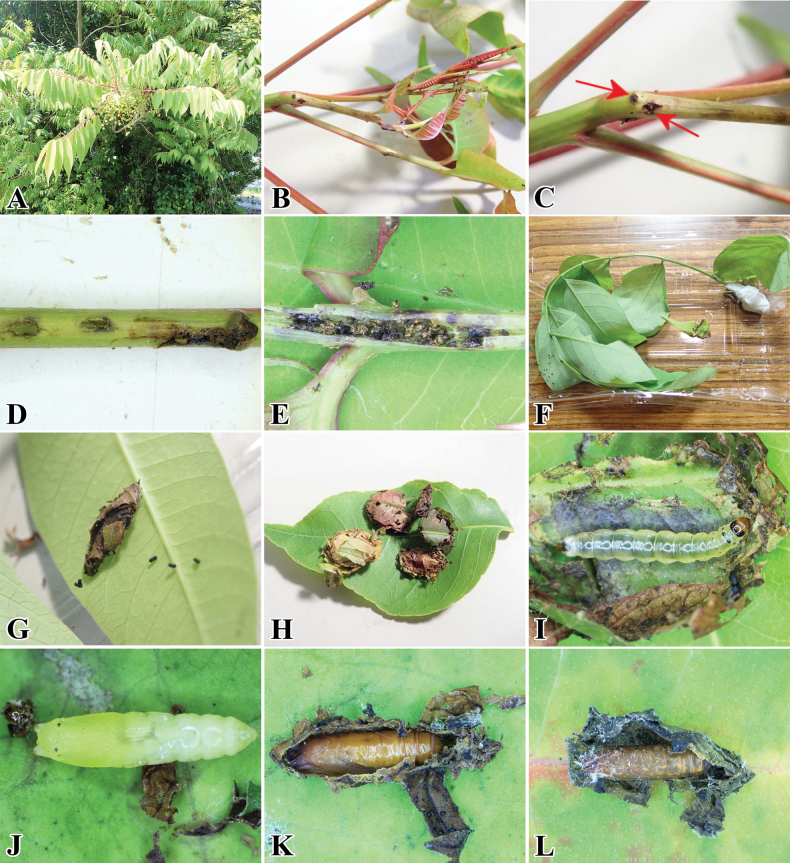
*Palumbinagrandiunca* and its host plant. **A** host plant: *Toxicodendronsuccedaneum* (Anacardiaceae) **B** infected young petiole **C** initial small hole on young petiole **D** small holes after the larva leave from the petiole **E** larva feces inside the infected petiole **F** leaf cut by larva **G** larval case attached to leaf surface **H** leaf cases **I** larva inside its leaf case **J** young pupa **K** pupa **L** pupal exuvia left by an emerged adult.

*Toxicodendronsuccedaneum* (L.) Kuntze (Anacardiaceae) (Fig. 12A).

##### Biology.

(Fig. 12A–L). First, the larva mines the petiole of its host plant and later makes a portable leaf case after leaving its mine. At the early instar stage, the larva first made a small hole to enter the petiole (Fig. 12B–D). After that, it started to mine, and larval feces were deposited inside the mine (Fig. 12E). After the larva gradually grows and develops into the late instar, it leaves from its mine (Fig. 12D) and then cuts the leaf into small irregularly shaped pieces (Fig. 12F). Later, many small leaf pieces were aggregated and stacked into a compact and circular leaf case. The larvae live and feed inside the case and can move from one place to another by carrying it (Fig. 12G, H); pupation also occurs inside it (Fig. 12J). The adult emerges by leaving the pupal exuvia inside the case (Fig. 12K). The resting posture of the adult was similar to that of the stathmopodid species, keeping its hindlegs upwards (Fig. 9C).

##### Remarks.

This species has already been recorded as *Palumbina* sp. 1 (Oku et al. 2018) on Amamioshima Island in Japan.

#### ﻿ Palumbina operaria

Taxon classificationAnimaliaLepidopteraGelechiidae

(Meyrick, 1918)

AEB36750-6F54-50A7-B87B-B7B96DC64ED8

Figs 2A–F , 6D , 7D , 8D Japanese name: Midare-mongin-hoso-kibaga

Thiotricha operaria Meyrick, 1918: 125; Meyrick, 1925a: 103; Clarke, 1969: 463; Ivinskis et al. 1984: 39. TL: Assam, India. TD: NHMUK.
Palumbina operaria : Lee et al. 2018: 29, fig. 84.

##### Material examined.

Japan – **Honshu** [Mie] • 1♀; Nabari-shi, Kaochidani 280 m; 28 Jun. 2015; S. Yagi leg.; ELKU. – [Yamaguchi] • 1♂, 1♀; Kawakami, Hagi, Chomonkyo; 10 Sep. 2013; S. Yagi leg.; gen. slide no. KM–389; ELKU. – **Shikoku** [Ehime] •1♀; Narukawa-keikoku, Kihoku, Kitauwa-gun; 2 Sep. 2017; S. Yagi leg., gen. slide no. *Palumbina* No. 1; ELKU. – [Kochi] • 1♂; Kuroson; 18 Jun. 1964, S. Moriuti leg.; OPU. – **Kyushu** [Kagoshima] • 1♂, 1♀; Yakushima Is., Oko-Rindo 600m; 29 Jul. 2013; T. Terada leg.; KGU. • 1♂; same locality; 21 Jun. 2017, LT; S. Yagi leg.; gen. slide no. KM–313; ELKU. – **Ryukyus** [Okinawa] • 4♂♂; Ishikagijima Is., Mt. Omotodake; 15 Apr. 1962; Y. Arita leg.; gen. slide no. KM–314, 315; NSMT. • 1♂, 1♀; same data except 4 May 1978; NSMT. • 1♂; same data except 5 May 1978; gen. slide no. KM–316; NSMT. • 1♀; Ishikagijima Is, Mt. Bannadake; 7 May 1998; T. Ueda leg.; gen slide no. KM–317; OPU. • 2♀♀; same data except 4–7 Apr. 2001, gen. slide no. KM–318; OPU. • 1♀; Iriomotejima Is., Taketomi-cho, Mt. Komi; 13 Mar. 2011; T. Terada leg.; KGU. • 1♂; Iriomotejima Is., Funaura; 27 Mar. 2002; T. Hirowatari leg.; OPU. • 1♂, 2♀♀; Iriomotejima Is., Taketomi-cho, Uehara; 12 Mar. 2011; T. Terada leg.; KGU. • 2♂♂; Iriomotejima Is., Mt. Tedou, Uehara; 8 Jul. 2017, LT; S. Yagi leg.; ELKU.

For the diagnosis and detailed description of the adults and genitalia, see Lee et al. (2018).

##### Host plant.

Unknown.

##### Biology.

The adults were collected from March to September.

##### Distribution.

Japan (Honshu, Shikoku, Kyushu, Ryukyu), China and India.

##### Remarks.

This species has interspecific wing marking variations (Fig. 2A–F).

#### ﻿ Palumbina macrodelta

Taxon classificationAnimaliaLepidopteraGelechiidae

(Meyrick, 1918)

3B4AE5A9-8E5B-5F0E-9A0E-215FB7E40615

Figs 3A, B , 6E , 7E , 8E Japanese name: Futamon ginhoso-kibaga

Thyrsostoma macrodelta Meyrick, 1918: 121; Meyrick 1925a: 100; Gaede 1937: 301; Clarke 1969: 484. TL: Assam, India. TD: NHMUK.
Palumbina macrodelta : Sattler 1982: 25; Lee et al. 2018; 17, fig. 71.

##### Material examined.

Japan – **Ryukyus** [Kagoshima] • 1♂; Amamioshima Is., Uken-son, Mt. Akatuti; 3 Mar. 2012; S. Sameshima leg.; gen. slide no. KM–325; KGU. – [Okinawa] • 3♂♂; Ishigakijima Is., Mt. Nosoko-dake, 25–29 Mar. 2020; S. Tomura leg.; gen. slide no. SY1455, SY1456, *Palumbina* No. 4; ELKU. • 3♂♂; Ishigakijima Is., Mt. Omoto-dake; 2 May 1978; Y. Arita leg.; gen. slide no. KM–323; NSMT. • 3♂♂, 1♀; same data except 17 Nov. 1980; gen. slide no. KM–326 (♀); NSMT. • 1♂, 1♀; same locality; 1 Nov. 1979; A. Nakayama leg.; gen. slide no. KM–327(♀); NSMT. • 1♂; Ishigakijima Is., Mt. Banna-dake; 3 Nov. 1979; A. Nakayama leg.; gen. slide no. KM–324; NSMT. • 1♀; same locality; 3 Nov. 1979; M. Yamashita leg.; NSMT. • 1♂; same locality; 7 May 1998, T. Ueda leg.; OPU. • 1♂, 2♀♀; same locality; 4–7 Apr. 2001, T. Ueda leg.; OPU. • 1♂; Ishigakijima Is., Mansei-rindo; 30 Mar. 2020; S. Tomura leg.; gen. slide no. *Palumbina* No. 3; ELKU. • 6♂♂, 1♀♀; Iriomotejima Is., Funaura; 21–25 Mar. 1995; T. Mano leg.; OPU. • 1♀; Iriomotejima Is., Uehara (Taketomi-tyo); 24 Oct. 2000; T. Saito leg.; OPU. • 1♂; same data except 3 Oct. 2001; OPU. • 1♀; Iriomotejima Is., Mt. Tedou, Uehara; 8 Aug. 2017, LT; T. Hirowatari, S. Yagi & K.M.M. Kyaw leg.; gen. slide no. KM–328; ELKU.

For the diagnosis and detailed description of the adults and genitalia, see Lee et al. (2018).

##### Host plant.

Unknown.

##### Biology.

The adults were collected from March to November.

##### Distribution.

Japan (Ryukyus), China (Lee et al. 2018), and India.

#### ﻿ Palumbina muraseae

Taxon classificationAnimaliaLepidopteraGelechiidae

Kyaw & Yagi sp. nov.

E7BD0381-0515-5261-BB38-A088E2A49EA1

https://zoobank.org/EFE89CFE-F7D0-46E1-906E-C85590DC7C12

Figs 3C–F , 4 , 5 , 6F , 7F , 8F , 9D , 13 , 22 , 23 Japanese name: Murase-mongin-hoso-kibaga

Thyrsostoma sp.: Murase 2009: 140, figs 13–17.
Palumbina sp. 2: Oku et al. 2018: 32, fig. 47.

##### Type material.

***Holotype***: Japan – **Ryukyus** •♂; Kagoshima Pref., San Tokunoshima; 9. Jul. 2016; Sadahisa Yagi leg.; gen. slide no. KM–374; ELKU. ***Paratypes***: Japan – **Kyushu** [Kagoshima] • 2♂♂; Yakusima Is., Hirauti; 19 Sep. 1978; Y. Arita leg.; gen. slide no. KM–342; NSMT. • 1♂, 1♀; Yakusima, Satunan Is.; 17 Oct. 1973; T. Kumata; gen. slide no. KM–343; SEHU. – **Ryukyus** [Kagoshima] • 3♂♂, 1♀; Amamioshima Is., Mt. Yuwan-dake; 15 Nov. 2012; S. Sameshima leg.; gen. slide no. KM–334(♂), 335(♂), 336(♂), 345(♀); KGU. • 1♀; same locality; 18 Nov. 2012; K. Tsuda & S. Sameshima leg.; gen. slide no. KM–349; KGU. • 1♂; same locality; 23 May 2014; gen. slide no. KM–341; KGU. • 1♀; same data except 5 May 2015; KGU. • 3♂♂, 2♀♀; Amamioshima Is., Uken-son, Ohata; 14 Sep. 2002; Host: *Distyliumracemosum*; T. Ueda leg.; 27 Sep–3 Oct. 2002 em.; OPU. • 1♂; Amamioshima Is., Mt. Akatuti, Uken-Son; 21 Sep. 2012; S. Sameshima leg.; gen. slide no. KM–337; KGU. • 2♂♂; Amamioshima Is., Mt. Akatuti; 19 Nov. 2012; K. Tsuda & S. Sameshima leg.; gen. slide no. KM–339; KGU. • 1♂; same data except 7 Mar. 2013; gen. slide no. KM–338; KGU. • 1♂; same data except 27 May 2015; gen. slide no. KM–340; KGU. • 2♂♂, 2♀♀; Amamioshima Is., Sumiyou-son, Yakukatsu; 12–15 Sep. 2002; Host: *Distyliumracemosum*; T. Ueda leg.; 27 Sep. – 3 Oct. 2002 em.; OPU. • 2♀♀; Amamioshima Is., Kamiya, Sumiyo-Son; 23 May 2015; S. Sameshima leg.; gen. slide no. KM–346; KGU. • 1♀; Tokunoshima Is., Yamakubiri-rindo, Tokunoshima-Town; 2 May 2015; Y. Sakamaki leg.; gen. slide no. KM–344; KGU. • 1♂; same data as holotype, ELKU. – [Okinawa] • 1♂; Okinawajima Is., Mt. Nishime, Benoki; 8 Aug. 2017, LT; S. Yagi, T. Hirowatari, K.M.M. Kyaw leg.; ELKU. • 1♂; Okinawajima Is., Kunigami-gun, Kunigami-son, Uka; 31 May 2015, LT; S. Yagi leg.; ELKU. • 1♀; Okinawajima Is., Benoki, Kunigami-son, Mt. Terukubi, 330m ; 4 Aug. 2015; S. Yagi leg.; gen. slide no. KM–348; ELKU. • 1♂, 1♀; Okinawajima Is., Kunigami-son, Yona; 23 Mar. 2002; Host: *Distyliumracemosum*; 13–15 Apr. 2002 em.; gen. slide no. KM-427(♀); OPU. •1♀; same data except; 24 Mar. 2002; OPU. • 1♀; same locality; 24 Mar. 2002; T. Saito leg.; OPU. • 1♂1♀; Okinawa-Is., Iji, Kunigami-Gun; 2 Jun. 2017 (320m); Y. Kitajima leg.; KGU. • 3♀♀; same locality, 25 Mar. 2021, LT (317m); S. Tomura leg.; ELKU. • 1♀; Okinawajima Is., Ookuni-rindo (Kunigami-son); 2 May 2000; T. Saito leg.; OPU. • 8♂♂, 3♀♀; Okinawa Is., Yaka, Kin-cho, Kunigami-gun, 25 Mar. 2021 (larva); Host: *Distyliumracemosum*; 15–21 Apr. 2021 em.; S. Yagi leg.; ELKU. • 1♀; Ishigakijima Is., Mt Yarabu, Sakieda; 5 Jul. 2017; S. Yagi leg.; ELKU. • 1♂1♀; Ishigakijima Is., Ishigaki-shi, Mt. Omoto; 18 Mar. 2002; I. Ohshima leg.; ELKU. •1♂; Ishigakijima Is., Ishigaki-shi, Funra, 15 Mar. 2002; I. Ohshima leg.; ELKU. • 1♀; Ishigakijima Is., Hirae, Oyamizu-hiroba; 4 Jul. 2017; S. Yagi leg.; gen. slide no. KM–347; ELKU.

**Figure 13.**
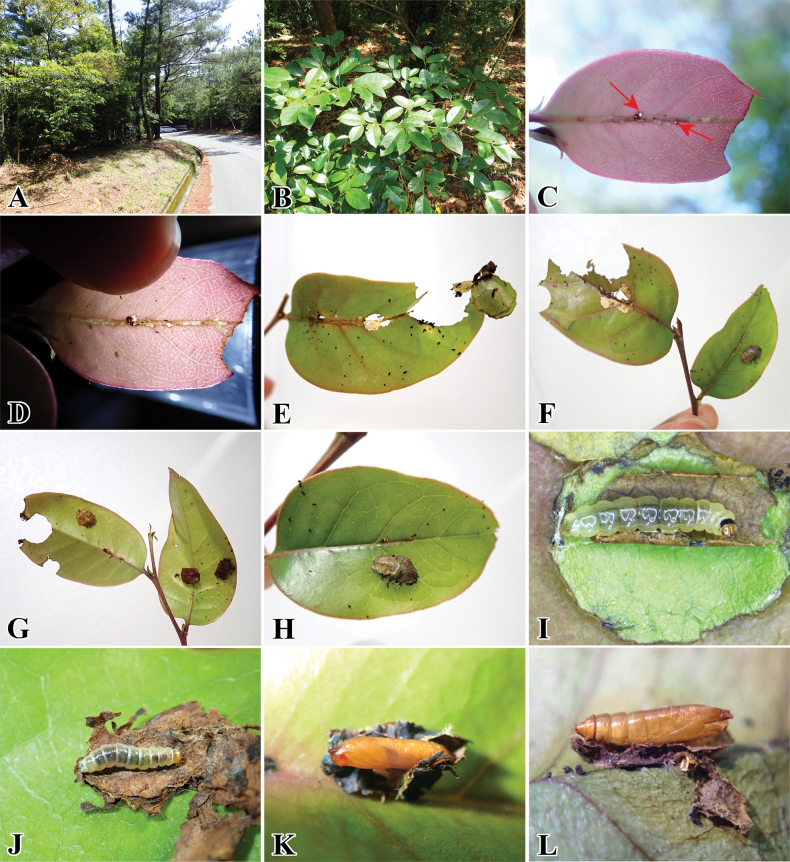
*Palumbinamuraseae* sp. nov. and its host plant. **A** habitat **B** host plant: *Distyliumracemosum* (Hamamelidaceae) **C** infected young leaf, arrow indicates small holes made by the larva **D** midrib of leaf mined by the larva and the larva feeding inside it (red square) **E** upper leaf tip cut by the larva **F** larval leaf case on the upper leaf surface **G** larval leaf case on the lower leaf surface **H** close up of larval leaf case **I** young larva inside the fresh leaf case **J** mature larva inside the dried leaf case **K** pupa **L** pupal exuvia left by an adult.

##### Diagnosis.

This species can be easily distinguished from other congeneric species by having a yellowish ocher or dark brown color with small white patches at the base and before the apex in the forewing, racket-shaped anellus lobes, uncus basally with a knob with numerous stout setae ventrally, extremely broad succus in the male genitalia, and rectangular process of signum in the female genitalia.

**Figure 14.**
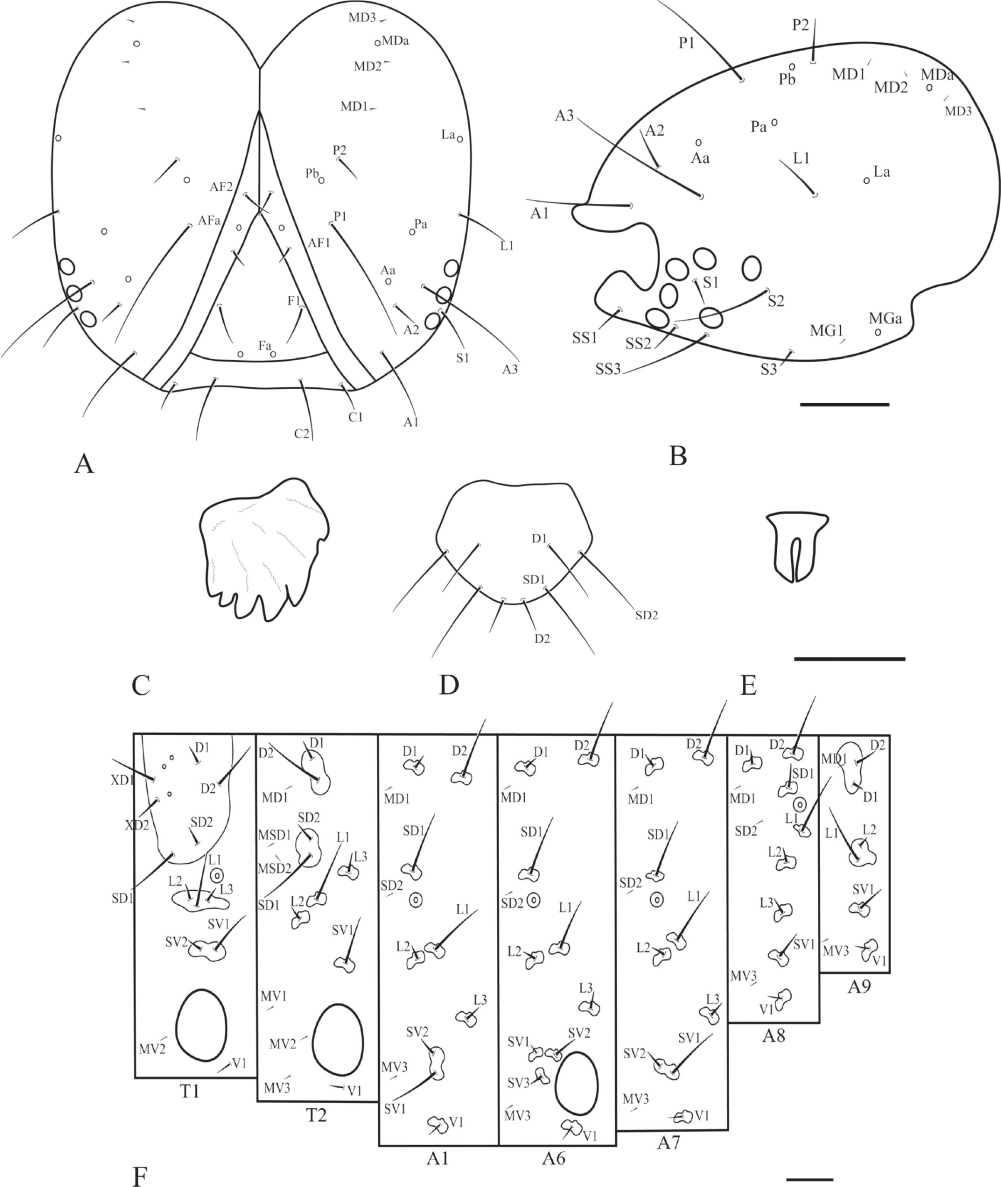
Chaetotaxy of *P.pylartis*. **A** head, frontal view **B** ditto, lateral view **C** mandible **D** anal plate **E** anal fork **F** the prothorax, mesothorax and abdominal segments 1, 6–9. Scale bars: 0.10 mm (**A, B, F**); 0.20 mm (**C, D, E**).

##### Description.

**Male** (Figs 3C, E, 4A–C, 5A–C, E, 6F, 7F).

***Head*.** glossy white to fuscous. Scape creamy-white to ocherous-white; flagellum fuscous; cilia as long as width (Fig. 4A); flagellomeres I and II usually not entirely separated (Fig. 5A, B). Labial palpus creamy white, recurved, sexually dimorphic and modified in male; stout, shorter than female; segment I with outer surface fuscous, shortest, and as thick as segment II; segment II with expansible hair pencils arising from furrow on the ventral surface, reaching sub apex of segment III (Fig. 4C); segment III as long as segment II, dorso-distal half fuscous. Proboscis scaly white.

**Figure 15.**
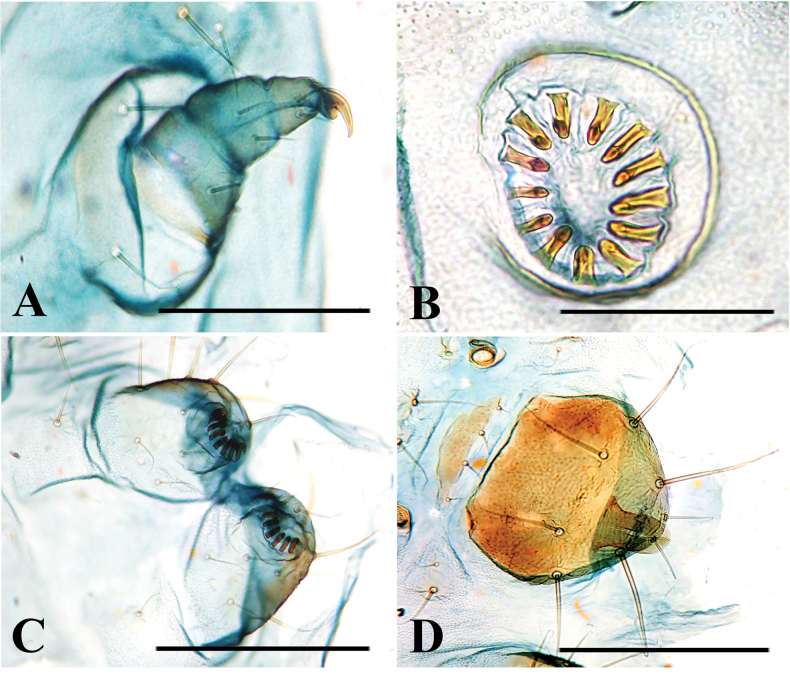
Larva of *P.pylartis*. **A** thoracic leg **B** crochets **C** anal proleg **D** anal shield. Scale bars: 0.40 mm (**A, C, D**); 0.20 mm (**B**).

***Thorax***. Dorsum of thorax and tegula white to yellowish ocher. Legs creamy white; fore femur creamy-white, fore tibia with fuscous on the outer surface and sometimes on the inner surface, fore tarsus black on the outer surface, each tarsomere ringed black apically on inner surface; mid-femur white on the outer surface and creamy yellowish on inner surface; mid-tibia creamy white with three black spots, mid tarsus creamy white; first tarsomere black at middle and apex, remaining tarsomeres ringed black apically; hind femur creamy white, hind tibia suffused with black at 1/3 and apex near tibial spur at ca. middle on outer surface; bearing long bristles along basal 2/3 of dorsal margin and with whorls of bristles at the apex (Fig. 4E); hind tarsus, mostly fuscous.

**Figure 16.**
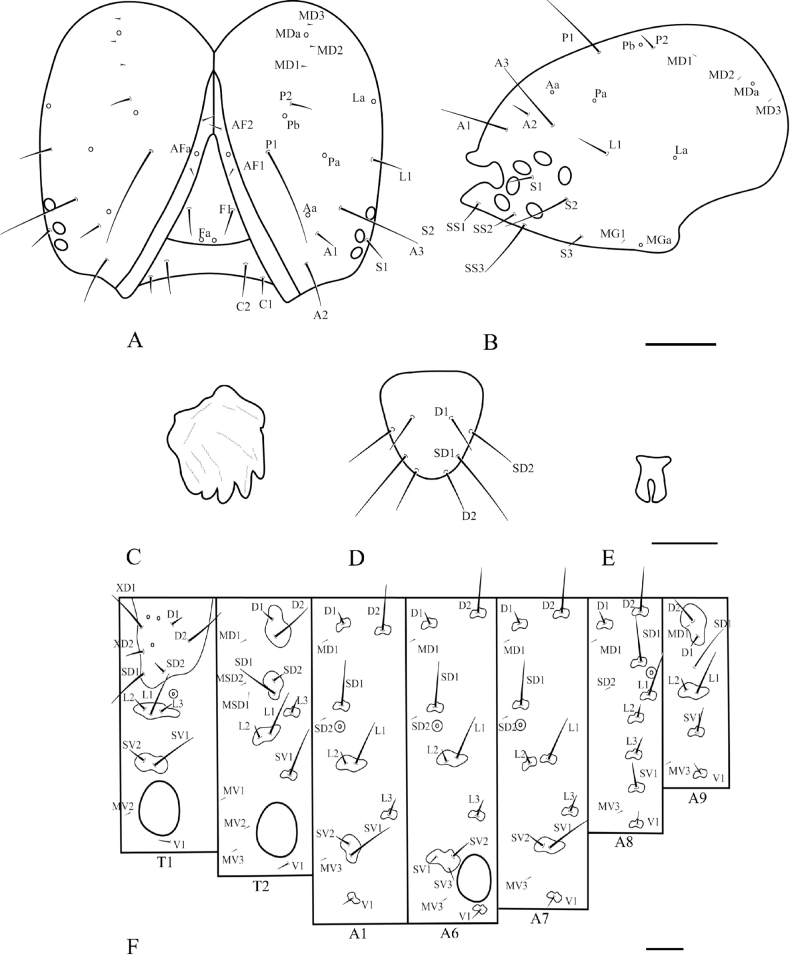
Chaetotaxy of *P.grandiunca*. **A** head, frontal view **B** ditto, lateral view **C** mandible **D** anal plate **E** anal fork **F** the prothorax, mesothorax and abdominal segments 1, 6–9. Scale bars: 0.10 mm (**A, B, F**); 0.20 mm (**C, D, E**).

***Forewing*.** Length 3.8 mm in holotype, 2.9–3.9 mm in paratypes (*n* = 9). Wing expanse 8.4 mm in the holotype, 6.1–8.5 mm in the paratypes (*n* = 9) (Fig. 3C, E). 11 veins: Sc reaching basal 1/2 of costa, R1 and R2 free, R3 and R4 short-stalked, R5 absent, M1 connate with R3+4, M2 remote from M3, CuA1 parallel to CuA2, CuA2 indistinct, 1A+2A forked at the base (Fig. 5E); ground color yellowish ocher to brown, mostly with yellowish ocher reflections in holotype, sometimes suffused with creamy white at ~ 1/4 near the base and ~ 1/4 before the apex, sometimes delineated with black scales throughout the costal margin from its base to ~ 1/2 forewing length before apex; expansible pale yellowish ocher hair pencils beneath costa on ventral surface (Fig. 4B); cilia well-fringed, yellowish ocher from its apex to tornus, yellowish white to the inner base of forewing.

**Figure 17.**
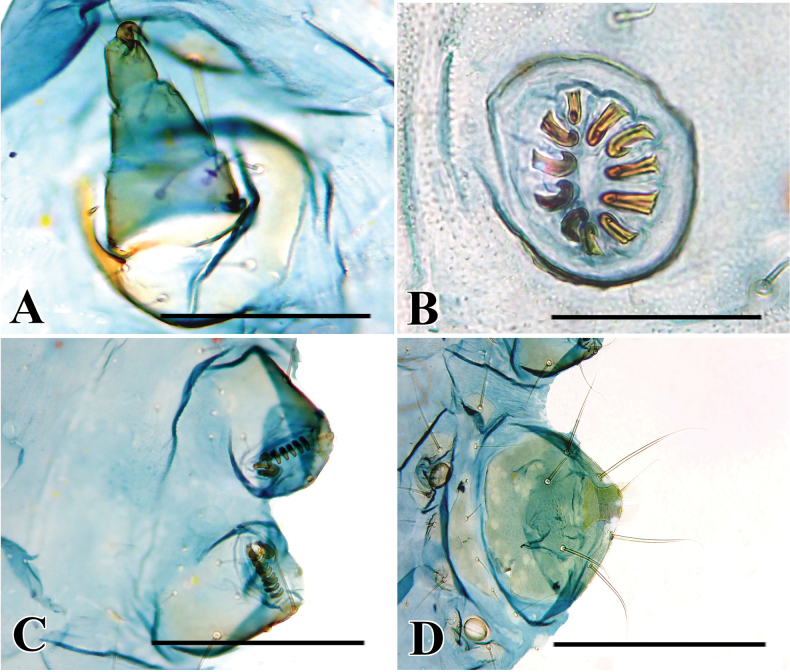
Larva of *P.grandiunca*. **A** thoracic leg **B** crochets **C** anal proleg **D** anal shield. Scale bars: 0.40 mm (**A, C, D**); 0.20 mm (**B**).

***Hindwing*.** Narrower than forewing, 8 veins, R1 join with Sc near the base, Rs and M1 stalked at distal 1/5, M2 remote from M3, CuA1 and CuA2 parallel (Fig. 5E), creamy white with yellowish brown margin from costal area to beyond tornus; with a row of bristles arising from basal 1/6 to 1/5 of costa; cilia well-fringed, yellowish brown throughout to tornus, yellowish white to the inner base of hind wing.

**Figure 18.**
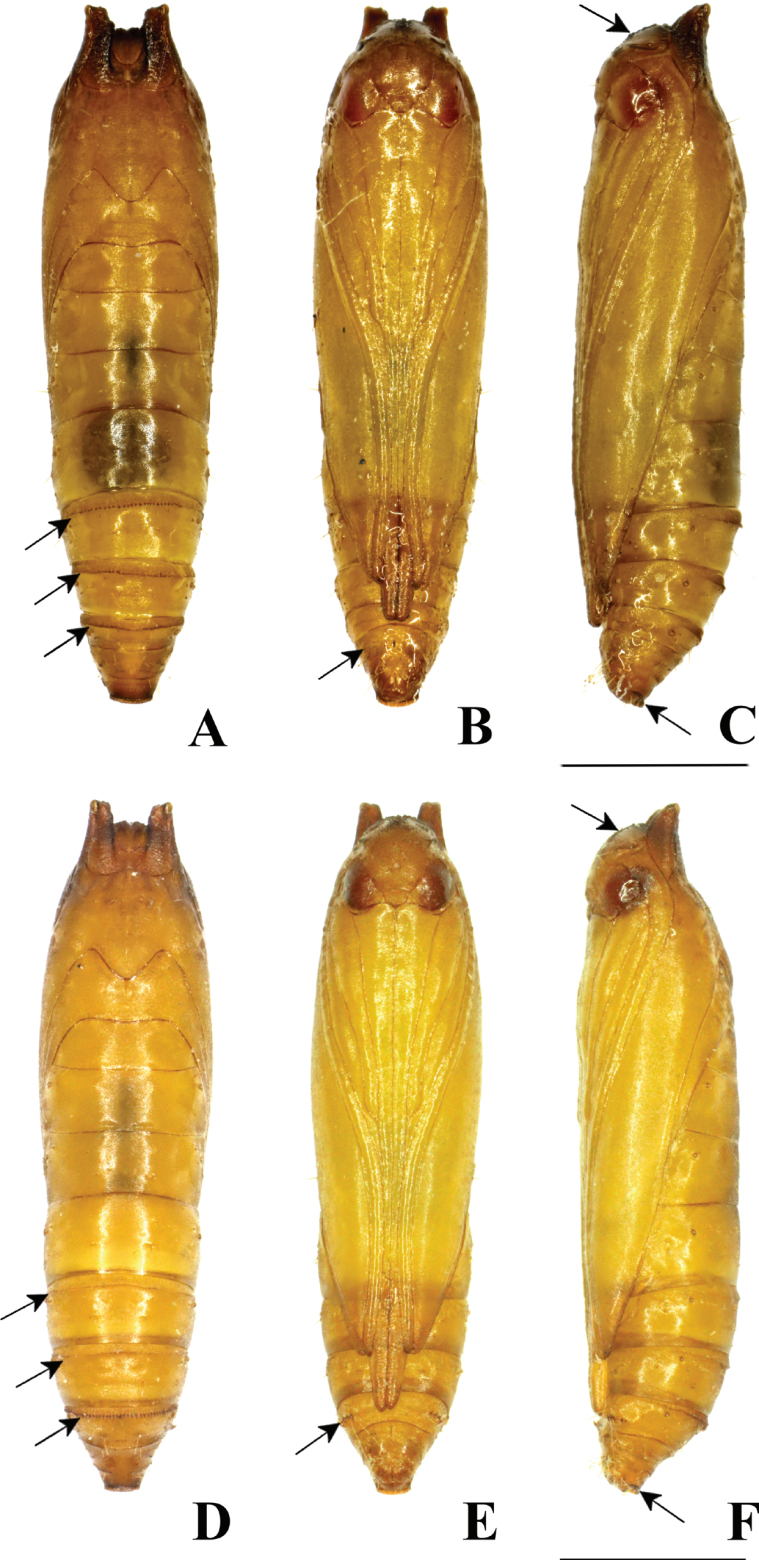
Pupa of *P.pylartis*. **A–C** male pupa **D–F** female pupa **A, D** dorsal view **B, E** ventral view and **C, F** lateral view. **A** arrows on A5, A6 and A7 indicate a transverse row of tergal spinules anteriorly **B** and **E** arrow on A7 indicates an oval pad without a row of spinules in males and with a row of spinules in females **C** and **F** the arrow on the vertex indicates many minute spines, and the arrow on A10 indicates short spinules **D** the arrow on A7 indicates a row of tergal spinules near anterior margin and arrows on A5 and A6 indicate a transverse row of dot-like spinules. Scale bars: 1.0 mm.

***Abdomen*** (Fig. 7F). Coremata absent. Terga and sterna 1–7 unmodified. Sternum 8 large, rounded with sclerotized margin, slightly daunted at the middle on posterior margin.

**Figure 19.**
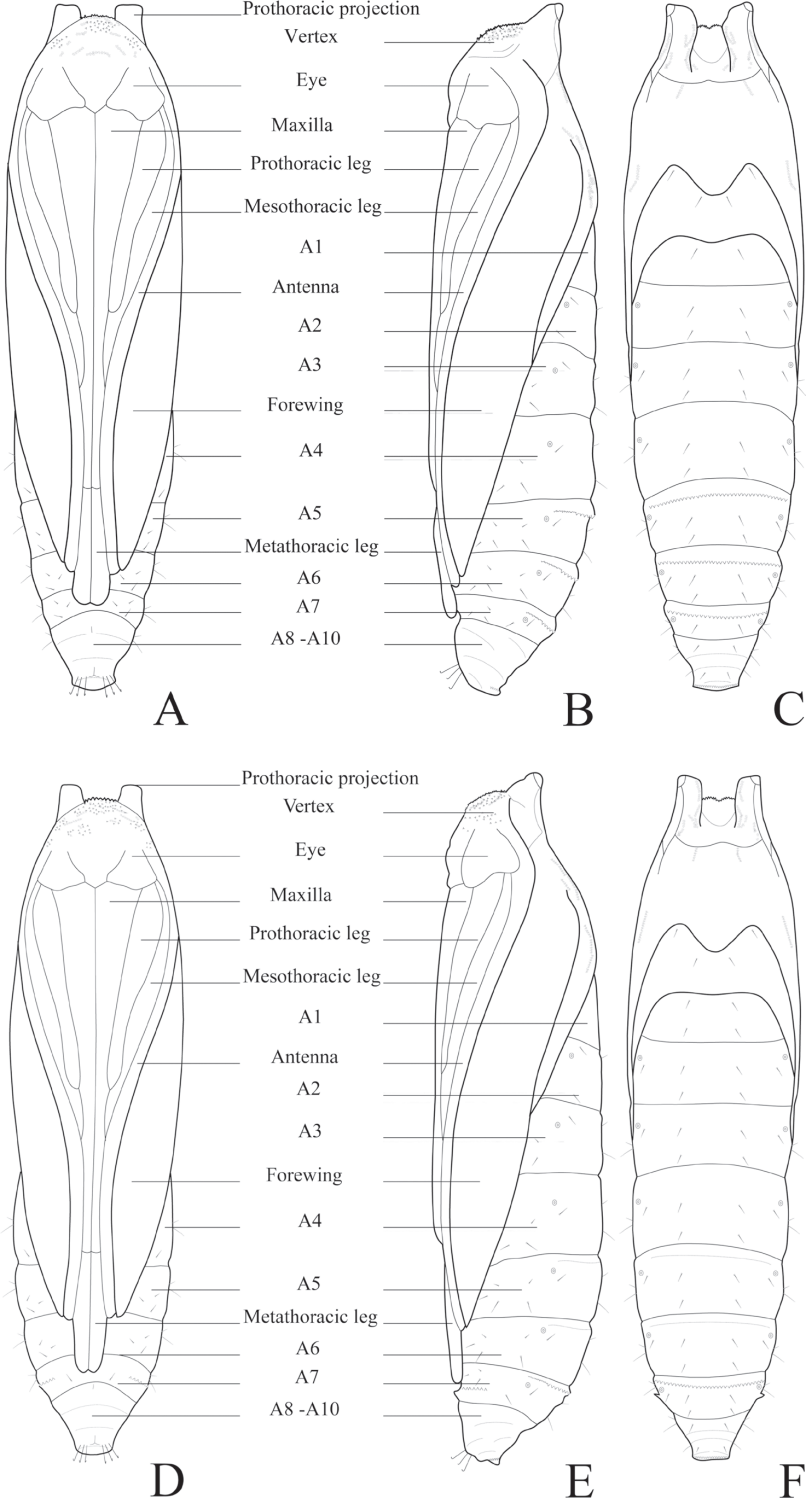
Pupa of *P.pylartis***A–C** male pupa **D–F** female pupa **A, D** ventral view **B, E** lateral view and **C, F** dorsal view.

***Male genitalia*** (Fig. 6F). Uncus fan-shaped, bearing a few setae on its dorsal surface, with a narrow furrow from its base to ~ 1/2 distance of its ventral surface, forming a long and small knob basoventrally, bearing numerous microtrichia ventrally with many stout setae apically. Culcitula present. Tegumen ~ 2.5× longer than uncus, broadly concave on the anterior margin. Gnathos hook long and recurved upwards, moderately stout at ~ 2/3 basally, taper towards apically with slightly pointed tip. Valva ~ 1.3× as long as tegumen, broadened basally, gradually narrowed to ~ 1/2 length, then elbowed at ca. its middle and slightly curved towards apex, uniformly elongated with rounded tip, densely setose with numerous short and long fine setae on the apical inner surface. Anellus lobe small and ~ 1/8 length of valva, racket-shaped with flexible long setae on the tip and numerous short setae on the ventral surface. Saccus extremely broad, sub-triangularly produced towards apex. Juxta with a pair of rather long and beak-shaped processes with pointed tip bearing fine setae apically. Phallus long, basal 1/4 dilated, 3/4 slender and sinuous, interior sclerite arising from its basal 1/4, nearly reaching the apex.

**Figure 20.**
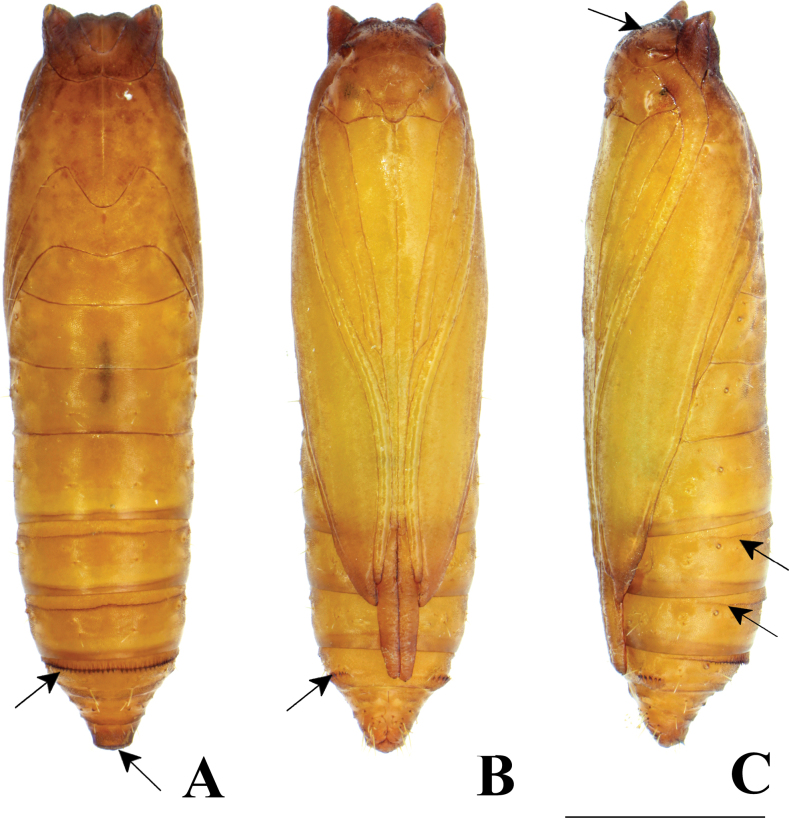
Female pupa of *P.grandiunca*. **A** dorsal view **B** ventral view **C** lateral view. **A** Arrow on A7 indicates a transverse row of tergal spinules anteriorly, and the arrow on A10 indicates short spinules. **B** the arrow on A7 indicates an oval pad with a row of spinules. **C** the arrow on the vertex indicates many minute spines, and arrows on A5 and A6 indicate a transverse row of dot-like spinules. Scale bars: 1.0 mm.

**Female** (Figs 3D, F, 4D, 5D). ***Forewing*.** Length 3.1–4.2 mm (*n* = 9), wing expanse 7.0–9.1 mm (*n* = 9).

**Figure 21.**
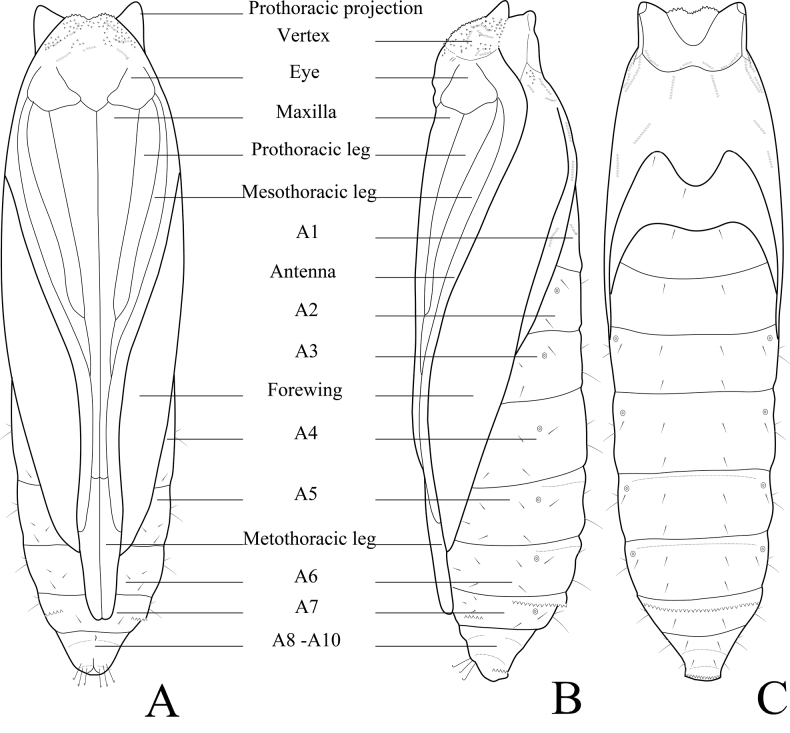
Female pupa of *P.grandiunca*. **A** ventral view **B** lateral view **C** dorsal view.

***Head*.** Similar to males, but it differs as follows: flagellum without ciliation, labial palpus slender; segment II dorso-distally fuscous; segment III brown to fuscous.

***Female genitalia*** (Fig. 8F). Papillae anales bilobed. Apophyses long. Apophysis anterioris ~ 3/5 length of apophysis posterioris. Tergum 8 divided into two sclerites, with their inner margins close posteriorly and remote anteriorly. Sternum 8 narrowly elongated and projected into funnel-shaped sclerotized structures. Antrum broad and heavily sclerotized. Ductus bursae moderately broad and short, dilated near antrum. Corpus bursae oblong; signum situated in the middle, basal plate more or less oblong with a sclerotized ridge medially on its surface, with a rectangular process inwardly bearing a row of teeth apically.

**Figure 22.**
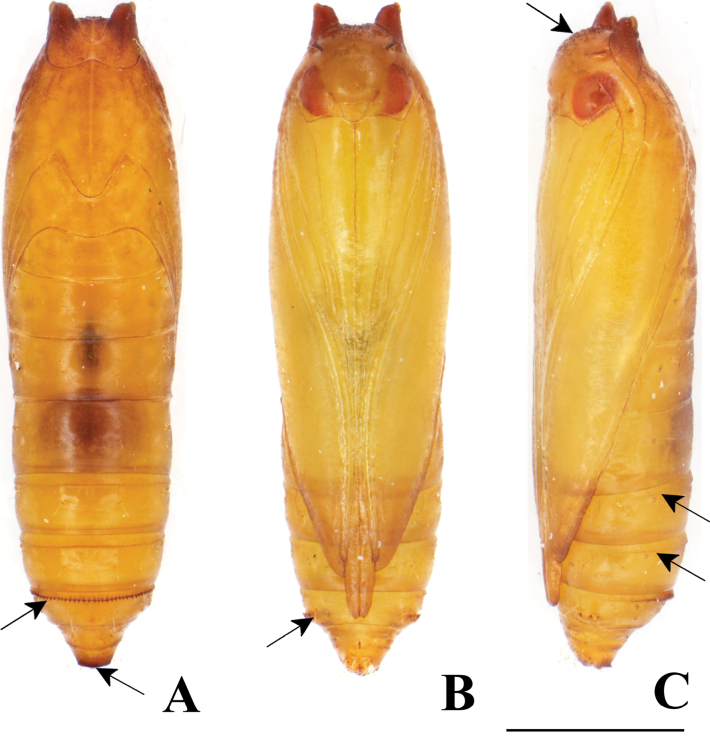
Female pupa of *P.muraseae* sp. nov. **A** dorsal view **B** ventral view **C** lateral view. **A** arrow on A7 indicates a transverse row of tergal spinules near anterior margin and arrow on A10 indicates short spinules **B** arrow indicates an oval pad equipped with a row of spinules **C** arrow on the vertex of the head indicates many minute spines arrows on A5 and A6 indicate a transverse row of dot-like spinules. Scale bars: 1.0 mm.

**Larva** (Fig. 13I). Length ~ 2.6–3.1 mm (*n* = 10), slender. Head semi-globular. Body pale yellow in early instars and yellowish brown with black pigmentation on ocellar area and on anterior margin of labrum in late instars. Prothoracic shield yellowish brown, blackish brown on caudal margin in later instars. Thoracic leg short, pale yellowish brown. Body creamy white. Pinaculum circular, blackish brown on T1–T3 and A1, A2, A8, and A9, and paler on remaining abdominal segments. Anal shield heavily sclerotized, yellowish brown. Anal fork present. Anal prolegs with many minute spines on the dorsal surface.

**Figure 23.**
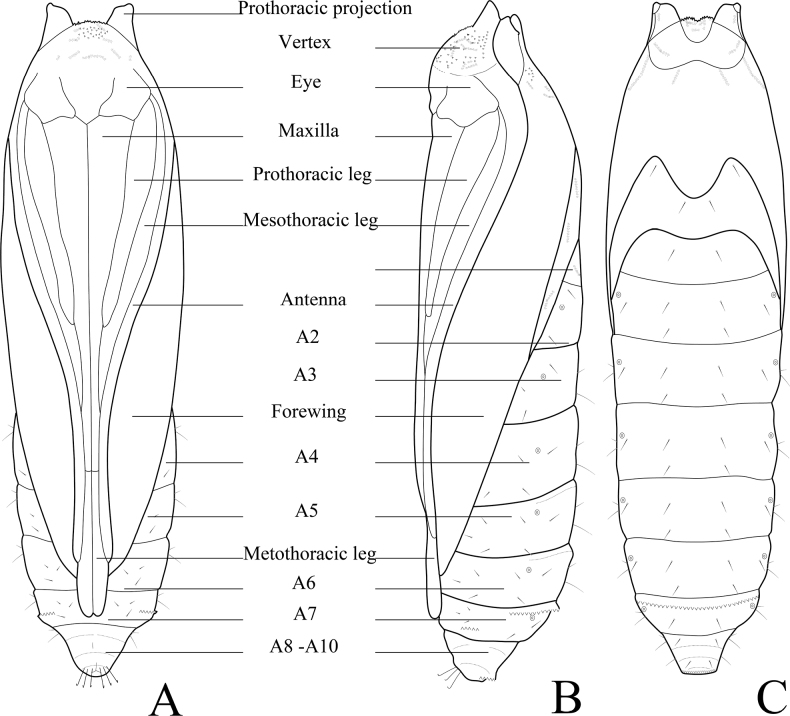
Male pupa of *P.muraseae* sp. nov. **A** ventral view **B** lateral view **C** dorsal view.

**Pupa** (Figs 22, 23). Length 3.5–4.2 mm (*n* = 4), cylindrical. Color yellowish, dark brown emergence. Head semi-globular. Vertex with many minute granular spines. Prothorax with a pair of more or less wedge-shaped projections on dorsolateral corners of tergite (Fig. 23A). Antennae extending to anterior margin of A6. Forewings extending to the middle of A6. Maxilla (galea) basally broad, gradually narrowing and extending to near posterior margin of A4. Prothoracic legs extending to near posterior margin of A2; mesothoracic legs extend to near posterior margin of A4; metathoracic legs extend to the posterior margin of A7. A5–A10 movable. A5 and A6 with a transverse row of dot-like spinules on the anterior margin (Fig. 22C). A7 with transverse tergal spinules directed anteriorly on the anterior margin (Fig. 22A). Sternite A7 with a pair of oval pads armed with a row of spinules directed anteriorly (Fig. 22B). A10 delineated with a row of short spinules at the outer margin posteriorly (Fig. 22A); apically with three pairs of hooked setae on ventral surfaces of A9 and A10; no true cremaster present.

##### Etymology.

The scientific name of the species is dedicated to Ms. Masumi Murase, who first collected the species and reported its biology.

##### Distribution.

Japan (Kyushu, Ryukyus).

##### Host plant.

*Distyliumracemosum* Siebold et Zucc. (Hamamelidaceae).

##### Biology

(Fig. 13). The larvae mine at the midrib of the leaf to complete their growth and development (Fig. 13C). The small holes seem to be used not only for entering the midrib but also for ejecting its feces. It leaves its mine after it gradually matures. After leaving, the larva cuts transversely at ~ 1/3 of the apical leaf and makes it into a shelter. The larva usually consumes leaves from its shelf around the radius it can reach. Eventually, the larva cut the leaf into a small and irregular shape to construct a portable case (Fig. 13E, F). Later, the larva repeats this process until pupation, accumulating and stacking these small leaf pieces into a compact and more or less circular shape (Fig. 13G, H). When the larva is close to pupating, it fixes its case and pupates in its case (Fig. 13K). The adult emerges and leaves the pupal exuvia inside (Fig. 13L). Murase (2009) pointed out that the resting posture of emerged adults is similar to that of stathmopodid species, keeping their hindlegs upwards, shown in Fig. 9D.

##### Remarks.

Murase (2009) reported this species as an undetermined species feeding on the host plant *Distyliumracemosum* and described larval feeding: making a portable case on that host plant, wherein the larva cuts the young leaves into a circular shape and then feeding inside the case. Subsequently, the larva moved with its case and fixed it to the lower surface of the leaf or the wall of the rearing container. Finally, the larvae were transformed into pupae inside the case. This species has already been recorded as *Palumbina* sp. 2 (Oku et al. 2018) on Amami Oshima Island in Japan.

## ﻿Discussion

### ﻿Larval feeding habits and shape of the portable case

Based on our observations of three *Palumbina*, the larval feeding habits of all examined species is not a direct case maker: they have two feeding phases in which the larva first attacks as a miner into the petiole or the midrib of its host plant; then, the larva makes its portable case and lives by feeding inside it. Such a two-phase feeding mode has been observed in *Calliprora* (Lee and Hayden 2019), and perhaps other species of the genera in Thiotrichinae also have such feeding modes.

Noticeably, it can be assumed that the larvae of *Palumbina* make a portable case by utilizing the leaf, although we could not reveal the biology of the remaining two Japanese species, *P.operaria* and *P.macrodelta*. This feeding habit making portable case has also been observed in *Thiotricha* and *Pulchrala* (Kyaw et al. 2019, 2021). However, in our study, the circular and flattened portable case was not observed in other Thiotrichinae species. On the other hand, larvae of *P.guerinii* (Stainton, 1858) were found in the galls of Aphididae or on fruits of the host plant (Sattler 1982), and their feeding habit might be different. Therefore, the circular and flattened portable case may be characteristic of only some *Palumbina* species.

### ﻿Larval chaetotaxy

The immature stages of *Palumbina* have not been reported in any previous studies. In larval chaetotaxy, the two examined species, *P.pylartis* and *P.grandiunca* are the same as those of general gelechiid larvae in having such characteristics: the distance between L1 and A3 is far from A3 to A2 on the head (Figs 14A, 16A); on the abdominal segment, an anal fork in A10 (Stehr 1987).

Compared with *Thiotricha* species (e.g., *T.prunifolivora* Ueda & Fujiwara, 2005, *T.lumnitzeriella* Kyaw, Ueda & Hirowatari, 2021, *T.gemmulans* Meyrick, 1931, and *Calliproraleucaenae* Lee & Hiden, 2019 (Ueda and Fujiwara 2005; Lee and Hayden 2019; Kyaw et al. 2021), the larval chaetotaxy of *P.pylartis* and *P.grandiunca* were mostly congruent with that of *Thiotricha*. However, in the head region of the two *Palumbina* species, AF1 is shorter than AF2, whereas in some *Thiotricha* species with equal length of AF1 and AF2 (Ueda and Fujiwara 2005; Kyaw et al. 2021). In *Calliproraleucaenae*, AF1 is longer than AF2 (Lee and Hayden 2019). The number and position of some setae in *P.pylartis* and *P.grandiunca* in the abdominal segments were similar to those of *Thiotricha*: D1 is always above D2 on A1–A3; the SV group is bisetose on A1 and A7 and tri-setose on A2–A6, and uni-setose on A8 and A9. However, in *Calliprora*, D1 is below D2 on A1–A3, the SV group is uni-setose on A1, A7–A9, and bi-setose on A2–A6 (Lee and Hayden 2019). Thus, *Palumbina* species seem to be more similar to *Thiotricha* species than to *Calliprora* species.

### ﻿Pupal morphology

The morphological characteristics of *P.grandiunca*, *P.muraseae* sp. nov., and *P.pylartis* are mostly shared with *Thiotrichaprunifolivora*, *T.lumnitzeriella*, and *T.gemmulans* (Ueda and Fujiwara 2005; Kyaw et al. 2021) as follows: projections at the anterolateral corner of prothorax developed; vertex with many minute spines; tergite A7 with a transverse row of tergal spinules directed posteriorly on the anterior margin and sternite A7 with a pair of oval pad armed with a row of spinules directed anteriorly. However, in the three *Palumbina* species, there are some exceptional characteristics in the abdominal segments of A7 and A10: the absence of a transverse row of short tergal spinules on A7 caudally and the absence of a pair of triangular tergal projections on A10.

Interestingly, the pupae of *P.pylartis* and *P.muraseae* sp. nov. (Figs 18–23) are sexually dimorphic in some characters on abdominal segments A5, A6, and A7. The male pupa possesses developed spinules directed posteriorly on A5 and A6; however, females have dot-like spinules on these segments. In sternite A7, male pupae do not possess a pair of oval pads armed with row spinules, but females possess such characteristics.

Such sexually dimorphic pupal morphology, except for the genital opening (slit), was noted in a few taxa. For example, *Phyllocnistiscitrella* Stainton, 1856 (Gracillariidae), wherein the last segment (presumably fusion of A9 and A10) bears two long hairs, but that of males exhibits a shorter pygidium without any hair (Jacas and Garrido 1996). Another example is *Tutaabsoluta* (Meyrick, 1917) (Gelechiidae): the male of this species has a longer case containing wings and legs; the case ends at A5 in females and A6 in males (Genc 2016). The adaptive significance of these differences is unknown, but sexual dimorphism of pupae may appear in more taxa.

In the three *Palumbina* species whose pupae are known, projections at the prothorax are prominent and may, thus, certainly be related to their mode of making a portable case similar to that of *Thiotricha* species (Ueda and Fujiwara 2005; Kyaw et al. 2019, 2021), not as in *Calliproraleucaenae* (Lee and Hayden 2019). It can be assumed that these projections may function to protrude to escape from its case, as in the genus *Thiotricha*. In contrast, many minute spines on the vertex of the head and A7 with a transverse row of tergal spinules dorsally shared within all known genera (*Calliprora*, *Palumbina*, *Thiotricha*, and *Pulchrala*) may be a synapomorphy of the subfamily Thiotrichinae. These similarities and differences may reflect the different feeding habits and shapes of cocoons.

### ﻿Resting posture of adults

Some studies (e.g., Murase 2009; Sakamaki 2013) have noted that the resting posture of the *Palumbina* species is similar to that of Stathmopodidae in keeping its hind legs upward. We observed the same resting posture in *Tenupalpa* species (e.g., *T.angustella* (Omelko, 1984) and *T.venustalis* (Omelko, 1984): Yagi pers. obs.). However, in *Pulchrala* species (e.g., *P.chujaensis* (Park, 2016) and *P.elaeocarpiella* Kyaw, Yagi & Hirowatari, 2019: Kyaw et al. 2019) and other Thiotrichinae species, the adults do not keep their hindlegs upwards when resting. Lee et al. (2021) suggested a close relationship between *Palumbina* and *Tenupalpa*, and “the hind-tibia with a posterior spur extending 2/3–3/4 length of tarsomere I” as one of the synapomorphies, while “as long or slightly exceeding half the length of tarsomere I” in other Thiotrichinae genera. The longer spur of the hind-tibia shared in *Palumbina* and *Tenupalpa* seems to be correlated with their adult resting posture, keeping their hindlegs upward, but the adaptive significance of this behavior is unclear.

## ﻿Conclusions

Lee et al. (2021) showed that *Palumbina*, *Thiotricha*, *Tenupalpa*, and *Pulchrala* formed a monophyletic group, but the phylogenetic relationships of this group remain controversial. Some shared traits among this group such as “the number of R veins is four in the forewing venation, the pupal morphology such as a pair of sub-triangular projections on the prothorax, and their biological traits of making a portable case” seems to indicate monophyly of this group. In addition, some unique characteristics, including the circular-shaped portable case and the morphology of the immature stages will be the key to revealing the detailed phylogenetic relationship in this group.

## Supplementary Material

XML Treatment for Palumbina

XML Treatment for Palumbina pylartis

XML Treatment for Palumbina acerosa

XML Treatment for Palumbina grandiunca

XML Treatment for Palumbina operaria

XML Treatment for Palumbina macrodelta

XML Treatment for Palumbina muraseae

## ﻿Additional information

### Author ORCIDs

Khine Mon Mon Kyaw https://orcid.org/0000-0001-5886-3376

Sadahisa Yagi https://orcid.org/0000-0002-4261-1219

Johei Oku https://orcid.org/0000-0001-9494-398X

Toshiya Hirowatari https://orcid.org/0000-0002-6839-2229

### Data availability

All of the data that support the findings of this study are available in the main text.
